# Ancient Expansion of the Hox Cluster in Lepidoptera Generated Four Homeobox Genes Implicated in Extra-Embryonic Tissue Formation

**DOI:** 10.1371/journal.pgen.1004698

**Published:** 2014-10-23

**Authors:** Laura Ferguson, Ferdinand Marlétaz, Jean-Michel Carter, William R. Taylor, Melanie Gibbs, Casper J. Breuker, Peter W. H. Holland

**Affiliations:** 1 Department of Zoology, University of Oxford, Oxford, United Kingdom; 2 Evolutionary Developmental Biology Research Group, Faculty of Health and Life Sciences, Department of Biological and Medical Sciences, Oxford Brookes University, Headington, Oxford, United Kingdom; 3 MRC National Institute for Medical Research, Mill Hill, London, United Kingdom; 4 NERC Centre for Ecology and Hydrology, Wallingford, Oxfordshire, United Kingdom; New York University, United States of America

## Abstract

Gene duplications within the conserved Hox cluster are rare in animal evolution, but in Lepidoptera an array of divergent Hox-related genes (Shx genes) has been reported between *pb* and *zen*. Here, we use genome sequencing of five lepidopteran species (*Polygonia c-album*, *Pararge aegeria*, *Callimorpha dominula*, *Cameraria ohridella*, *Hepialus sylvina*) plus a caddisfly outgroup (*Glyphotaelius pellucidus*) to trace the evolution of the lepidopteran Shx genes. We demonstrate that Shx genes originated by tandem duplication of *zen* early in the evolution of large clade Ditrysia; Shx are not found in a caddisfly and a member of the basally diverging Hepialidae (swift moths). Four distinct Shx genes were generated early in ditrysian evolution, and were stably retained in all descendent Lepidoptera except the silkmoth which has additional duplications. Despite extensive sequence divergence, molecular modelling indicates that all four Shx genes have the potential to encode stable homeodomains. The four Shx genes have distinct spatiotemporal expression patterns in early development of the Speckled Wood butterfly (*Pararge aegeria*), with *ShxC* demarcating the future sites of extraembryonic tissue formation via strikingly localised maternal RNA in the oocyte. All four genes are also expressed in presumptive serosal cells, prior to the onset of *zen* expression. Lepidopteran Shx genes represent an unusual example of Hox cluster expansion and integration of novel genes into ancient developmental regulatory networks.

## Author Summary

We have examined gene duplication in a set of ancient genes used in patterning of animal embryos: the Hox genes. These genes code for proteins that bind DNA and switch on or off other genes, and they are very similar between distantly related animal species. Butterflies and moths, however, have additional Hox genes whose origin and role has been unclear. We have sequenced the genomes of five species of butterfly and moth, and of a closely related caddisfly, to examine these issues. We found that one of the Hox genes, called *zen*, duplicated to generate four new genes in the evolution of the largest group of butterflies and moths. Further mutations greatly modified the DNA sequence of the new genes, although maintaining potential to encode stable protein folds. Gene expression also changed so that the new Hox-derived genes are deployed in egg and early embryonic stages marking the tissues that will later envelop, nourish and protect the embryo.

## Introduction

The characterization of Hox genes in the 1980s awakened the idea that there may be similar processes controlling body patterning in divergent animals and gave the first opportunity to compare the control of developmental processes between taxa at a molecular level. In animals as evolutionarily divergent as insects, annelids and vertebrates, Hox genes encode transcription factors deployed in early development, most notably to control spatial identity along the anteroposterior axis of the developing embryo [1].

Conservation of Hox gene function is reflected in their constrained evolution. First, there is high conservation of encoded protein sequence, particularly within the 60-amino acid homeodomain motif (encoded by the homeobox) containing three alpha helices. Second, Hox genes are often arranged in a genomic cluster, which was generated by tandem gene duplication early in animal evolution [2], [3]. Gene order is generally constrained, partly through shared and long-range regulatory elements [1], [4], [5]. Third, after expansion of the Hox cluster in early animal evolution there has been relatively little variation in gene number. The ancestor of all Ecdysozoa, Lophotrochozoa and Deuterostomia possessed 7 to 10 Hox genes [3], and most bilaterian animals still have approximately this number despite hundreds of millions of years of subsequent evolution. The lack of expansion of the Hox gene cluster within Bilateria is intriguing and is in contrast to the pattern of evolution seen for many other sets of genes [6], [7]. Exceptions are Hox cluster expansion to 15 genes in amphioxus [8], [9] and duplication of the entire gene cluster in vertebrates [2], [5], [10].

There are few recorded cases of tandem duplication *within* the Hox gene cluster. The best characterised example relates to the Hox paralogy group 3 (PG3) gene of insects, called *zerknullt (zen*), which has duplicated in a beetle (*Tribolium castaneum*) to yield *zen* and *zen2* [11], and in cyclorrhaphan flies to generate *zen* and the highly derived *bicoid (bcd)* [12]. A further duplication specific to the genus *Drosophila* generated *zen2* [13]. Furthermore, early in insect evolution the *zen*/PG3 gene lost its ancestral function of providing positional identity along the anteroposterior axis, and acquired a novel role in extra-embryonic tissue formation [14], [15], [16].

There are indications that the Hox gene cluster also expanded in Lepidoptera. Analysis of the Domesticated Silkmoth *Bombyx mori* genome revealed a large array of divergent homeobox genes, named Shx (Special homeobox) genes, between *pb* and *zen* [17]. With 12 Shx loci described, in addition to *zen*, the canonical Hox genes and another divergent gene *ftz*, the Silkmoth has the largest Hox gene cluster described [17]. The Silkmoth Shx sequences are highly divergent; some loci have internal duplications manifest as two or three homeobox sequences per gene, and some have disruptive mutations and are probably pseudogenes. The Hox gene cluster has also been characterised in the nymphalid butterflies *Heliconius melpomene* and *Danaus plexippus* (Monarch) where four homeobox genes were found between *pb* and *zen* [18], [19]. To date, the timing of the gene duplications, the ancestral condition for the Lepidoptera, variation in Shx gene number and gene expression have not been addressed.

Here we investigate the origin and evolution of *Shx* genes through sequencing and assembly of genomes from six species representing successively diverging lepidopteran lineages as well as an outgroup from Trichoptera (caddisflies). We find that four distinct Shx genes arose from the *zen* gene in the ancestor of the Ditrysia, the clade encompassing most Lepidoptera, and that this complement, not the expanded number found in *Bombyx*, is the norm across lepidopteran evolution. By modelling tertiary structure, we show that Shx protein sequence is compatible with folding into helix-loop-helix-turn-helix homeodomains. Finally, we determine the expression of Shx genes in early developmental stages of the Speckled Wood butterfly *Pararge aegeria*. These data suggest that Shx genes encode homeodomain proteins with probable roles in extra-embryonic tissue specification and formation. The lepidopteran *zen* gene may play a more downstream role in extraembryonic membrane function following serosal closure.

## Results

### Genome sequencing of Lepidoptera

**Figure 1:**
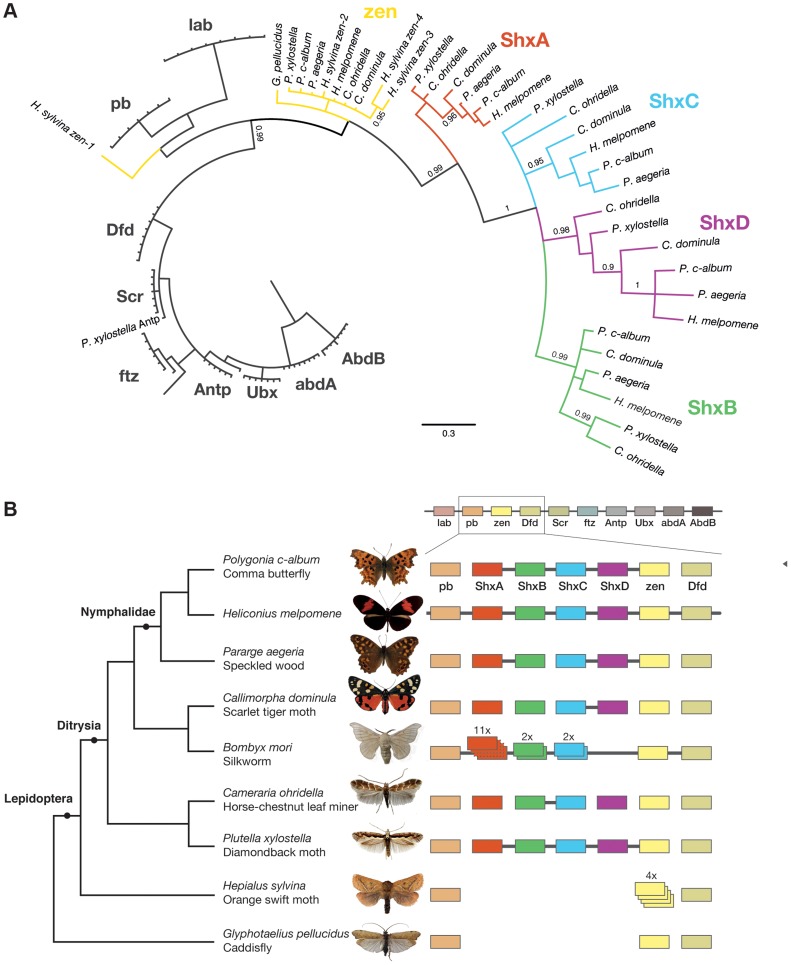
Shx genes originated as tandem duplications of *zen* within the Hox gene cluster. (A) Phylogenetic tree of Shx among lepidopteran Hox genes reconstructed using Phylobayes (C20), support values are posterior probabilies. (B) Shx complement of 8 lepidopteran species and the Trichoptera outgroup with available linkage information. Presence of multiple genes on the same genomic scaffolding is indicated by a plain line and gene duplication within a paralogy group as stacked boxes.

We generated low coverage genome sequences for six species chosen for their phylogenetic positions (Figure 1B). Shx sequence data were also extracted from genome projects of the Silkmoth [20], the Diamondback moth *Plutella xylostella* [21], and the butterflies *H. melpomene* [18] and the Monarch *D. plexippus* [19]. The last two species are members of the Nymphalidae, the largest butterfly family, which we elected to sample further using the Comma and Speckled Wood butterflies (*Polygonia c-album* and *Pararge aegeria*). To deduce the ancestral condition for the major ditrysian clade encompassing all butterflies and the majority of moths [22], [23], [24], we also selected the Scarlet Tiger moth *Callimorpha dominula* (family Arctiidae). To examine deeper in the evolutionary history of Lepidoptera, we chose the Horse Chestnut Leafminer moth *Cameraria ohridella* (family Gracillariidae) which, along with the Diamondback moth (Yponomeutoidea) represents one of the earliest evolutionary lineages of Ditrysia [21], [22], [23], [24]. As an outgroup to Ditrysia we selected the Orange Swift moth *Hepialus sylvina* (synonym *Trioda sylvina*, family Hepialidae), and for an outgroup to the Lepidoptera we used a caddisfly *Glyphotaelius pellucidus* (order Trichoptera). The Trichoptera and Lepidoptera together form the sister clade to the Diptera (flies).

Genomic DNA was sequenced using Illumina HiSeq technology, and multiple assemblies constructed using a range of k-mer sizes. For each species, we sequenced between 31.6 and 83.1 million paired-end reads granting coverage ranging from 6× to 17× as determined using a k-mer spectrum approach. We generated draft genome assemblies from 337 Mb to 1.4 Gb using de Bruijn approaches, yielding N50 values up to 5.3 kb. These datasets also provide the first estimates of genome size for these species (Table 1). Since our goal was gene and homeobox sequence hunting, rather than large-scale synteny analysis, relatively low N50 sizes are sufficient. To determine if the coverage generated was suitable, we searched the assemblies for the canonical Hox genes (*lab, pb, Dfd, Scr, Antp, Ubx, abdA, AbdB*) and *ftz*. All Hox genes were identified for all species, apart from the homeobox of Orange Swift *Ubx*, affording confidence in our sequencing approach to identify novel *Hox* genes in non-model lepidopteran species. In order to confirm that we did not lose genes during assembly of the raw read data, we also applied an alternative assembly strategy that maximally includes all sequence reads. This did not reveal any additional homeobox sequences.

**Table 1:** Summary statistics of genome sequencing and assembly.

Common Name	Scientific name	PE reads	Total reads	Insert size	Best k-mer	Assembly size	N50	Estimated coverage	Estimated genome size (Mb)
Caddisfly	*Glyphotaelius pellucidus*	44,410,472	88,820,944	370	51	757,289,448	1467	8.12	757
Orange Swift Moth	*Hepialus sylvina*	83,154,465	166,308,930	370	41	1,328,286,773	836	5.83	1791
Horse Chestnut Leafminer Moth	*Cameraria ohridella*	54,039,161	108,078,322	250	51	345,888,571	1783	16.21	474
Scarlet Tiger Moth	*Callimorpha dominula*	50,419,378	100,838,756	250	41	564,766,187	2567	12.80	666
Speckled wood Butterfly	*Pararge aegeria*	52,535,837	105,071,674	320	51	422,678,431	5441	17.06	477
Comma Butterfly	*Polygonia c-album*	31,612,456	63,224,912	370	51	405,525,838	2548	10.59	451

We were able to reconstruct genomic scaffolds around the Shx, *zen, pb* and *Dfd* genes by manually inspecting and aligning contigs from multiple assemblies, enabling the definition of gene models spanning multiple exons, as well as confirmation of linkage between adjacent genes in several species (Figure 1, Table S1).

### Evolutionary origins of Shx genes

To examine the gene duplication events that generated Shx genes, we used molecular phylogenetic analysis and comparison of gene content between different species. Homeodomain phylogenetic trees demonstrate that the Shx genes form a monophyletic group (BP 86, PP 0.99) and are more closely related to *zen* than to any other Hox gene (Figure 1A, Figure S1). This suggests that Shx genes originated by tandem duplication from an ancestral *zen* gene, consistent with their genomic location between *pb* and *zen* (Figure 1B). Sequence alignments incorporating conserved domains outside the homeodomain confirmed this result (Figure S2).

In phylogenetic analyses, Shx genes divide into four distinct orthology groups each present in Speckled Wood, Comma, Scarlet Tiger moth, Horse Chestnut Leafminer and the Diamondback moth. The *ShxA, ShxB, ShxC* and *ShxD* groups identified in the butterflies *H. melpomene* and Monarch therefore originated in the clade Ditrysia, which radiated 100 to 140 Myr ago and encompasses the vast diversity of lepidopteran species [25], [26]. The identity of putative *ShxC* genes of the Diamondback moth and Horse Chestnut Leafminer is not clear when only the homeodomain is used, but the existence of conserved motifs outside the homeodomain strongly argues for orthology with *ShxC*, as does overall protein sequence similarity, gene linkage and phylogenetic analysis with an extended alignment (Figure 1B, Figures S2, S3). Our re-analysis of the Silkmoth genome identifies the previously reported *Shx1* to *Shx11* [17], plus four additional homeodomain-containing open reading frames which fall within the ShxA and B clades and lie between *pb* and *zen*, here named Shx13-16 (Figure 1, Figure S4). This observation contrasts with the stability of Shx genes through most of ditrysian evolution.

We also investigated the Hox complement in the Orange Swift Moth, an outgroup to Ditrysia but within Lepidoptera, and the caddisfly (order Trichoptera), the sister order to Lepidoptera. We find the Orange Swift moth has no *bona fide* Shx genes, but several copies of *zen* gene that do not branch within established Shx groups in our phylogenetic analysis (Figure 1, Figure S1 and S3). Three (*zen2*, *zen3* and *zen4*) cluster with lepidopteran *zen* genes while *zen1* has a more ambiguous affinity (Figure 1, Figures S1 and S3). Presence of diagnostic motifs C-terminal to the homeodomain suggests all are duplications of *zen* (Figure S2 and Figure S5G). It is less probable that they share a common origin with Shx, with extensive divergence causing ambiguity of orthology assignment. Exons coding for the homeodomains plus a single probable 5′ exon of a *zen* gene are located on separate scaffolds that could not be linked.

The absence of *zen* duplication before lepidopteran radiation was confirmed by recovery of only a single *zen* gene in the caddisfly genome. Duplication and divergence of *zen* is therefore independent in Lepidoptera and Diptera.

### Evolution of lepidopteran zen and Shx gene sequence

Shx homeodomains have undergone faster sequence change than homeodomains encoded by *zen* or the canonical Hox genes. Homeodomain sequence of lab, pb, Dfd, Scr, Antp, Ubx, ftz, abdA and AbdB have 97% to 100% invariant sites across the four ditrysian Lepidoptera genomes sequenced in this study, canonical zen has 98% invariant sites and ShxA, ShxB, ShxC and ShxD have only 83%, 55%, 38% and 38% invariant sites respectively. Although lepidopteran *zen* and Shx genes are paralogues, and both descend from an ancestral *zen*, we retain the name Shx established in *Bombyx* [17] to reflect the more extreme sequence divergence in their homeodomains and to avoid confusion with earlier work. A number of conserved sites within the homeodomain are retained in Shx and zen, and S10 has been identified as unique to Hox3 orthologues (Figure S5I, red boxes) [15]; however, outside the homeodomain Shx proteins are radically different from each other and from zen (Figure S2, Figure S5C–F).

All lineages of ditryisian Lepidoptera (except *Bombyx*) have maintained a consistent complement of four different *Shx* genes, in addition to canonical *zen*, suggesting the genes have distinct functions. We examined whether gene-specific functions might be reflected in distinct protein motifs. Shx proteins have several short conserved motifs C-terminal to the homeodomain; these are different between the four proteins suggesting they may interact with different co-factors (Figure S2, Figure S5C–G). Lepidopteran zen shows more extensive protein conservation between species; these motifs are non-overlapping with those of the dipteran zen. Furthermore, analysis of caddisfly shows that motifs shared between basal Diptera and caddisfly have been lost in the Lepidoptera (Figure S2, Figure S5G, H). Rapid sequence evolution between closely related insect orders is consistent with a previous observation that outside the homeodomain there are no well conserved sequence motifs in *zen* genes of insects [27].

To investigate the dynamics underpinning diversification of Shx genes, we tested for signatures of selection by comparing synonymous (d_S_) and non-synonymous (d_N_) rates of substitutions in the homeobox region of Shx, zen and Hox genes in a maximum-likelihood framework. These analyses confirmed that there is strong purifying selection acting on the zen homeodomain in Lepidoptera (d_N_/d_S_ or ω = 0.002) comparable to that inferred for canonical Hox genes (ω ratio of 0.001). However, the Shx genes show a marked increase in coding substitutions with a d_N_/d_S_ ratio of 0.06; ShxB (ω = 0.1), ShxD (ω = 0.09) and ShxC (ω = 0.05) show more coding divergence than *ShxA* (ω 0.02). Accordingly, an excess of non-synonymous substitution is detected on the branch leading to the *ShxB, ShxC* and *ShxD* clade with an inferred ω ratio greater than 1 suggesting an episode of positive selection (Figure S6). We compared substitution ratios among codons within Shx proteins to determine whether some amino acids show evidence of positive selection. Using a site-model applied to Shx homeodomains only, we found an increased ω ratio at some sites but no statistical support (Table S2). However, taking the *zen* outgroup into account, the branch-site model found significant support (2*Δ*ℓ = 4.94, p<0.05) for positive selection at five sites (BEB pp>0.95). These sites are located between alpha helices and not known to be functionally involved in protein-DNA interaction (Table S2).

### Predicted structure of Shx homeodomains

**Figure 2:**
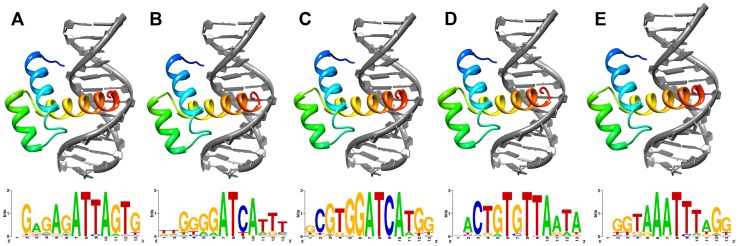
Lowest energy structural models of deduced (A) ShxA, (B) ShxB, (C) ShxC, (D) ShxD and (E) zen homeodomains from Speckled Wood *Pararge aegeria* bound to DNA sequences predicted through *in silico* evolution. Sequence logos generated from DNA sequences of 50 lowest energy predicted protein-DNA complexes for each protein.

As the Shx homeodomains have diverged extensively from the ancestral zen sequence, we asked whether they had undergone disabling mutations that might prevent them forming stable tertiary folds compatible with binding DNA. We deployed homology modelling based on a well-resolved experimentally-determined tertiary structure of a related Hox protein: that of the *Drosophila* Antp homeodomain bound to a 13-mer DNA sequence. Using the Comma and Speckled Wood butterfly sequences of ShxA, ShxB, ShxC, ShxD and zen, we first computed the native energy of the deduced structures modelled on the known Antp protein structure. Each yielded a stable predicted helix-loop-helix-turn-helix structure typical of a homeodomain (Figure 2), although stability was lower when modelled in complex with the specified 13-mer DNA sequence (Note S1). This suggests that the DNA sequence used was not optimal for these homeodomains.

To find more suitable DNA sequences, we used an *in silico* evolution approach and applied this to protein sequences of Comma, Speckled Wood and Horse Chestnut Leafminer, plus *Drosophila* Antp as a control. Starting with homopolymeric runs of either A, C, G or T, we ran 1000 cycles of ‘mutation’ and ‘selection’ to find the most energetically stable complexes, and generated consensus DNA sequences representing predicted optimal DNA binding sites for each homeodomain (Figure 2; Note S1). The evolved consensus sequence generated for *Drosophila* Antp was an approximation of the known DNA motif including the core ATTA which contacts with helix 3 of the homeodomain, plus a G residue immediately 5′.The evolved preferred DNA sequences for ShxA, ShxB and ShxC proteins included core ATTA or ATCA motifs, while the ShxD homeodomain showed more variation between the species preferring GTTA, ATTA or TTTA (Figure 2; Note S1). The zen proteins are somewhat different, tolerating a T in position 4 of the core. These results indicate that Shx and zen proteins have potential to fold into stable helix-loop-helix-turn-helix motifs compatible with sequence-specific DNA-binding. These analyses may not predict the exact in vivo binding sites [28], [29].

### Expression of Shx genes

**Figure 3:**
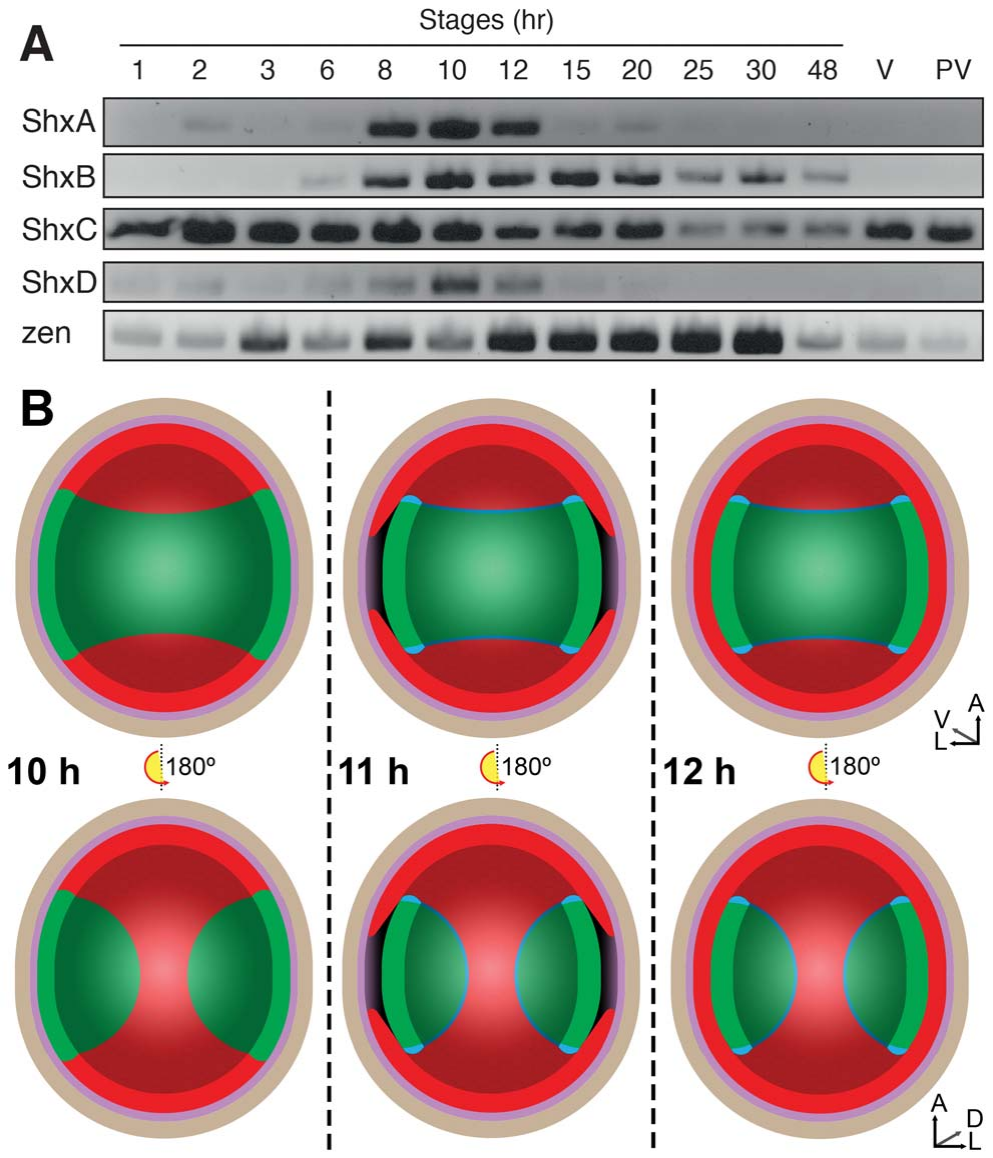
(A) Expression of Shx genes throughout embryonic stages of *P. aegeria*. Agarose gel electrophoresis of RT-PCR products obtained using intron-crossing primers (Figure S10). (B) Schematic overview of serosa formation in *P. aegeria*. Diagrammatic cross section through a developing embryo and associated extra-embryonic cell layers inside a 10–12 h AEL egg. Chorion (brown), vitelline membrane (violet), extraembryonic region/serosa (red), germ anlage (green) and presumptive amniotic cells (blue) are illustrated during serosal specification, maturation and closure. Top row shows ventral half while bottom row shows dorsal half, anterior is top in both. Embryo-vitelline cavity following germ anlage sinking is shown in the middle panel. Orientation 3D axis indicates anterior (A), left (L) and ventral (V) or dorsal (D). AEL, after egg-laying (hours).

During insect oogenesis, localisation of RNA derived from maternal gene expression establishes the future positions of embryonic and extra-embryonic regions within the oocyte, as well as its body axes (for an overview of lepidopteran embryology, see Kobayashi *et al.* [30]). Maternal transcripts of *zen* and *ShxC* (and weakly *ShxD*) were detected by RT-PCR in ovarioles dissected from Speckled Wood female imagos (Figure 3A). Consistent with this, we also identified these transcripts in a maternal transcriptome dataset [31] (ShxC:PaContig23051, GB:GAIX01013843.1, GI:509161192; ShxD:PaContig8659, GB:GAIX01015570.1, GI:509158266). After egg-laying (AEL) each Shx gene has a distinct temporal expression profile (Figure 3A). Our observations and comparison with other lepidopteran species [32], [33] suggests the onset of blastoderm cellularization and major zygotic transcription commences around 8 h AEL; expression of all four Shx genes plus *zen* is clearly detected between 8 and 12 h AEL.

**Figure 4:**
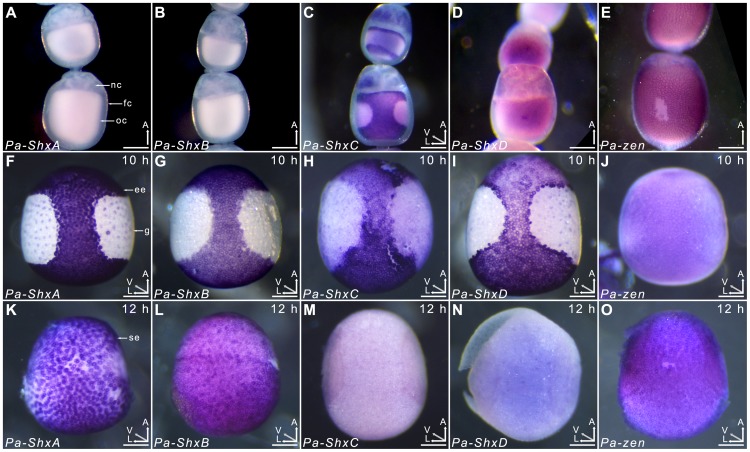
Spatiotemporal expression of Shx genes. Localisation of ShxA, ShxB, *ShxC*, *ShxD*, and *Pa-zen* transcripts in *P. aegeria* ovarioles (**A–E**), 10 h embryos (**F–J**) and 12 h embryos (**K–O**). Embryos and oocytes are orientated with the anterior to the top. Embryos dorsal side facing while lower and upper oocytes in **C** show dorsal and ventral faces respectively. Note that in 12 h embryos the serosal cells have migrated over the germ anlage forming an enveloping layer. Some follicle cells in **E** are removed to show absence of staining in the oocyte. Labels indicate nurse cells (**nc**), follicle cells (**fc**), oocyte (**oc**), germ anlage (**g**), and extra embryonic anlage (**ee**) which differentiates into the serosa (**s**). Orientation for each panel is indicated in bottom right 3D axis indicating anterior (A), left (L) and ventral (V) when known. AEL, after egg-laying (hours). Scale bars 200 µm.

*In situ* hybridisation to dissected ovarioles revealed that the spatial distribution of maternal *ShxC* and *ShxD* RNA is quite different to that of transcripts from their progenitor gene, *zen* (Figure 4). Pre-fertilisation transcripts from *ShxC* are detected in the nurse cells connected to the oocyte and are concentrated in a novel and striking asymmetrical ‘hourglass’ pattern which excludes the region later fated to become embryonic tissue, and corresponds to the presumptive serosal membranes (Figures 3B and 4C, Figure S7A–C). In contrast, transcripts of *ShxD* are faintly distributed throughout the developing oocyte without clear subcellular localisation (Figure 4D) and *zen* transcripts are specifically detected in the follicle cells surrounding the oocyte (Figure 4E).

In the embryo at 10 h AEL, transcripts of *ShxA*, *ShxB, ShxC* and *ShxD* are each detected in clear hourglass patterns in the cellularised blastoderm matching the earlier maternal *ShxC* RNA location in the oocyte (Figure 4F–I; Figure S7E–I). The location of Shx transcripts thus marks a clear distinction between the future embryonic regions (‘germ anlage’: small cells lacking *Shx* expression) and extraembryonic regions (larger cells expressing *Shx* genes). Within this latter domain, transcripts of *ShxD* are detected most strongly in the extraembryonic cells bordering the germ anlage (Figure 4I; Figure S7E–F, H–I). At the anterior pole of the egg near the micropyle, a cluster of cells with an increased concentration of *ShxD* transcripts correspond to a small region that previously lacked maternal *ShxC* transcripts (Figure S7D–F). In comparison, *zen* transcripts at 10 h AEL are very weakly detected throughout the blastoderm (Figure 4J).

Between 10 and 12 h AEL, the extraembryonic region expands over the germ anlage forming a protective serosal cell layer between the germ anlage and the vitelline membrane (Figure 3B). During this cell movement, *ShxC* and *ShxD* transcript levels, already lowered in the anterior (Figure S7E, F and I), reduce dramatically throughout the serosal layer (Figure 4M and N). However transcripts of *ShxA* and *ShxB*, which are only of zygotic origin, continue to be detected predominantly in the serosal layer, even after it has enveloped the germ anlage (Figure 4K,L). Transcripts of *zen* are detected in the serosa for the first time at this stage (Figure 4O) showing that expression patterns of *zen* and the *Shx* diverge dramatically in both time and space during butterfly embryogenesis. Significant zygotic transcription of the *ShxA* and *ShxD* genes was also detected in the large yolk cells beneath the blastoderm at 10–12 h AEL where transcripts were restricted to the nuclei suggesting either incipient transcription or RNA degradation in cytoplasm (Figure 4F,I; Figure S7H–J).

## Discussion

The common ancestor of living arthropods most likely had 10 Hox genes arranged in a single genomic cluster: *lab, pb, zen, Dfd, Scr, ftz, Antp, Ubx, abdA* and *AbdB* [3]. The primary roles of Hox genes in bilaterian animals, including arthropods, are to encode positional information and to instruct position-specific cell fate along the anterior posterior axis of the embryo. Two clear exceptions are *ftz*, which evolved a role in parasegment formation in insects, and *zen*. The evolutionary history of insect *zen* has been well studied. In chelicerates and a crustacean the orthologous gene has a typical Hox gene expression pattern [34], [35], while during insect evolution the gene diverged in sequence and acquired a different expression pattern and developmental role [14]. In addition to loss of Hox-like function, the *zen* gene of insects has undergone independent tandem duplications in the Flour Beetle (to yield *zen* and *zen2*) and the cyclorrhaphan flies (to yield *zen* and *bcd*) [12], [14]. In the *Drosophila* clade, within the Cyclorrhapha, *zen* has duplicated again to yield *zen* and *zen2* [36], [37].

*Zen* expression has been studied for a range of pterygote insects, including the Desert Locust *Schistocerca gregaria*, the Milkweed Bug *Oncopeltus fasciatus* [27], the Flour Beetle [38], and the flies [39]. Expression of the *Hox3/zen* precursor has also been analysed in an outgroup to the Pterygota, the apterygote Firebrat *Thermobia domestica* [40]. To some extent, inference of ancestral states within the insects is complicated by interspecific variation in the structure and function of the extraembryonic membranes and progression of embryogenesis [27]. In all pterygote insects studied however, *zen* expression is confined to the extraembryonic tissues with a dominant expression domain associated with early zygotic specification of the serosa, which in some species is accompanied by later, weaker expression in the amnion [14], [27], [38], [41].

Where *zen* duplication has occurred, both sub- and neofunctionalisation has occurred. Whereas zygotically expressed *zen* functions in extraembryonic membrane specification in *Drosophila*, maternally expressed *bcd* has radically diverged in sequence, and functions as an anterior determinant in the oocyte [12], [39]. A subsequent *Drosophila zen* duplication resulted in a putatively dispensable *zen2* paralog [36], unlike in the Flour Beetle where early-acting *zen-1* mainly specifies the serosal membranes and late-acting *zen-2* coordinates the fusion of amnion and serosa, initiating dorsal closure [38].

In the present study, we demonstrate that the *zen* gene duplicated during evolution of the Lepidoptera, independently of its duplication in Diptera and Coleoptera. In the Ditrysia, a clade encompassing most of lepidopteran diversity, these duplications generated four distinct Shx genes located next to the ancestral *zen* gene. Lepidopteran *zen* and Shx genes are co-orthologues of the ancestral *zen* gene, hence *ShxA* to *ShxD* could logically be called *zen2* to *zen5*. We retain the term Shx to avoid contradiction with earlier work, and to reflect their extensive sequence divergence and their shared ‘hourglass’ expression pattern in the blastoderm suggesting common functional roles. Additional Shx duplications occurred in the silkmoth lineage, but we find these are not typical of Lepidoptera. In the Orange Swift moth (Hepialidae), which diverged from a more basal node in lepidopteran phylogeny, Shx genes are not present but there is evidence of independent *zen* gene duplication. These data indicate that the generation of four recognisable Shx genes from an ancestral *zen* gene occurred after the Ditrysia had diverged; the common ancestor of Ditrysia and Hepialidae may have had multiple copies of *zen* but none had acquired sequence characters of Shx genes. The common ancestor of Lepidoptera and Trichoptera had just a single *zen* gene. The Shx genes are therefore an evolutionary novelty of ditrysian lepidopterans.

It is striking that all these examples of tandem gene duplication within insect Hox clusters can be traced to the same progenitor gene, *zen*. Indeed, we find no evidence of duplication of any other Hox gene within the Lepidoptera, and no such event has been reported in another insect. Why should the *zen* gene be prone to tandem gene duplication? The answer is likely to lie in the transition from an embryonic to extraembryonic function in the insects. If genomic clustering is important to Hox gene function, through shared enhancers or long-range chromatin effects, then tandem duplication of a canonical Hox gene would most likely disrupt regulation and generate a dominant effect mutation. Conversely, the expression of *zen* in extra-embryonic structures probably relies on a distinct regulatory mechanism less integrated with that of neighbouring genes; the immediate effect of duplication may therefore simply be increase of transcript dosage. The functional redundancy that is generated then offers potential for subsequent mutations to modify expression of either, or both, daughter genes.

After origin of the Shx genes, in an ancestor of the Ditrysia clade, the genes diverged radically in sequence, both within and outside the homeodomain. Within the Lepidoptera, the Shx genes also show an accumulation of coding substitutions, compared to other Hox genes, which likely reflects episodes of positive selection on some sites. In particular, we detect evidence of positive selection after the initial Shx gene duplicated to give *ShxA* and a progenitor of *ShxB*, *ShxC* and *ShxD*. We also find no Shx pseudogenes (except in the atypical *Bombyx*), but instead retention of the core set of these genes. Together these observations argue for functional constraints on Shx proteins and the acquisition of new essential roles for these genes in the biology of ditrysian lepidopterans.

Sequence divergence in the homeodomain raised the question of whether Shx proteins are still capable of functioning as DNA-binding proteins, potentially regulating the expression of other genes. Evidence that this biochemical role has most likely been retained comes from molecular modelling. We show that despite the extensive accumulation of amino acid substitutions in Shx homeodomains, they still have potential to fold into stable helix-loop-helix-turn-helix motifs with appropriate interaction surfaces for binding to DNA. An *in silico* evolution approach revealed that the Shx and zen proteins may have subtly different DNA sequence binding preferences, though these are not likely to be grossly dissimilar from target sequences recognised by canonical Hox proteins. We stress that these *in silico* approaches do not reveal definitive binding sites [28], [29]; however, they give confidence in the assertion that Shx proteins in Lepidoptera are likely to act as DNA-binding proteins.

What roles might Shx genes play in lepidopteran biology? Embryonic development is similar in the Silkmoth [42] and the Small White butterfly *Pieris rapae* suggesting conservation across the Ditrysia [30]. Following egg-laying the fertilised egg (zygote) undergoes continuous mitotic divisions and in the Silkmoth two regions can be distinguished in the cellular blastoderm based on cell density: the germ anlage which will become the embryo, and the remaining cells which will form the extraembryonic tissues notably the serosa [30], [42]. As observed for the Speckled Wood butterfly in the current study, in the Small White and Silkmoth, the presumptive serosa has a distinctive hourglass-shape [30]. At 10 h AEL in the Speckled Wood extraembryonic cells become polyploid, large and flat, and by 12 h this sheet of presumptive serosal cells moves over a region where more compact embryonic cells begin to sink into the yolk in the interior of the egg [32]. Serosal closure completes around 12 h AEL in the Speckled Wood butterfly (summarised in Figure 3B, Figure S8). As the embryonic germ anlage grows, cells at the edge of the anlage differentiate into a second extraembryonic membrane, the amnion, which extends around the ventral surface [30], [42].

The expression pattern of lepidopteran *zen* is intriguing because it differs from other insects. In Pterygota, except the Milkweed Bug, *zen* functions in early embryogenesis in the early specification of the extraembryonic membranes [14], [16], including in those species with a *zen* gene duplication. In the Lepidoptera, we find *zen* has largely lost this association and is instead expressed in follicle cells and then in the serosa following closure. Lepidopteran *zen* is therefore likely to have derived roles in the downstream functions of the serosal membrane. For example, we note that as the Speckled Wood *zen* expression intensifies, the maturing serosa takes on a glossy appearance indicative of cuticle secretion [43]. It has been suggested that the serosa plays roles in the innate immune system, processing environmental toxins, yolk catabolism, cuticle formation and desiccation resistance [44], [45].

The contrast between *zen* and *Shx* gene expression is striking. Our data reveal that Shx genes have a close association with development of the extraembryonic tissues of the Speckled Wood butterfly, but the *zen* gene does not. Indeed, all four *Shx* genes are expressed in the presumptive serosa well before *zen* expression is observed. We suggest that following *zen* gene duplication in Lepidoptera, the divergent Shx genes retained an ancestral association with extraembryonic membrane specification, while *zen* gene function diverged radically.

It would be a mistake, however, to consider all four lepidopteran Shx genes equivalent, as they have diverged from each other in sequence and in spatiotemporal expression patterns. Most strikingly, in the Speckled Wood there is maternal expression of *ShxC* and *ShxD*, but not *ShxA* and *ShxB*. It is notable that *zen* is maternally expressed in Locust and some basal fly species [39], [41], whilst in other pterygote insects *zen* transcripts are zygotically-derived. Maternal expression of *ShxC* and *ShxD* suggests that maternal expression may be an ancestral property of the *zen* gene [41]. However, in the flies and Locust *zen* transcripts are diffusely distributed within the oocyte, whereas in the Speckled Wood maternally-derived *ShxC* transcripts are tightly localised in a very distinctive hourglass shape, clearly prefiguring the region where extraembryonic tissues will later emerge after cellularisation. This hourglass pattern of *ShxC* transcripts within the single cell represents one of the most complex examples of RNA localisation ever reported in any species, and suggests that the Shx genes specify the future serosal tissue domain within the unfertilised oocyte. Differences between Shx gene expression domains are also seen in the embryonic stages: expression of *ShxC* and *ShxD* in serosal tissue is joined by expression of *ShxA* and *ShxB*, before these two genes become the dominant expressed Shx genes after serosal cell movements around the embryo.

The evolution of *Shx* genes provides some parallels to the evolution of *bcd* in Diptera. In both cases, the *zen* gene has undergone tandem duplication, daughter genes have diverged in sequence and there has been recruitment to patterning roles in the unfertilized oocyte.

## Materials and Methods

### Genome sequencing

DNA was extracted from individual adult specimens of the Comma butterfly (*Polygonia c-album*), the Speckled Wood butterfly (*Pararge aegeria*), the Scarlet Tiger moth (*Callimorpha dominula*), the Orange Swift moth (*Hepialus sylvina*) and a caddisfly (*Glyphotaelius pellucidus*), and from 75 pooled specimens of the Horse Chestnut Leafminer moth (*Cameraria ohridella*) using a phenol-chloroform method [46]. Sources of specimens are given in Table S3. Paired-end libraries were constructed and sequenced by Oxford Genomics Centre (http://www.well.ox.ac.uk) using standard Illumina procedures (http://www.illumina.com). Between 32 million and 83 million 101 bp paired-end reads were collected for each species (Table 1) using HiSeq2000 methodology. Low quality scoring bases were trimmed using sickle (https://github.com/najoshi/sickle.git). We assembled the reads using *de Bruijn*-based packages Velvet [47] and ABySS [48] with k-mers ranging from 31 to 61. Table 1 reports assemblies with the best combination of N50 and assembly length; these are available from the Oxford University Research Data Archive (DOI: 10.5287/bodleiandury.3). Alternative assemblies were also examined to assist with scaffolding around particular genes. As an additional method to identify homeodomain sequence contained in the reads, we also performed assembly using Fermi that implements an overlap-layout consensus approach using a FM-index and is designed to preserve all information in the raw reads [49]. Raw sequencing data are deposited in the NCBI BioProject database under accession number PRJNA241175. Genome size was determined using the k-mer spectrum approach: the frequency of all possible k-mers of a given length were calculated and plotted to reveal a peak representing the k-mer coverage (C_k_), while low and high k-mer coverages correspond to sequencing errors and repeated regions respectively (Figure S9). K-mer coverage was converted to actual base coverage (C) using C_k_ = C×(L−k+1)/L where L is the read length and k the k-mer size. K-mers were counted and distributions calculated using Jellyfish [50] for a k-mer size of 17 (Table 1).

### Hox gene identification

Analysis of the previously sequenced genomes of *Bombyx mori*, *Heliconius melpomene* and *Plutella xylostella* used data from Silkdb (http://silkworm.genomics.org.cn/), Butterflygenome (www.butterflygenome.org), KONAGAbase (http://dbm.dna.affrc.go.jp/px/) and the NCBI genome database. Scaffolds corresponding to the region *pb*-*Dfd* were downloaded and annotated according to conserved amino acid translations, sequence alignments and, where available, species-specific EST traces. Genome assemblies generated in this study were searched using the Hox homeodomains of *H. melpomene, B. mori* and *P. xylostella* using tBLASTn, scaffolds corresponding to significant hits (1e-6) were extracted and redundant scaffolds dismissed. Gene identification used a combination of phylogenetic analysis and amino acid signatures inside and outside the homeodomain (see below). Contigs containing homeoboxes were manually extended by generating a scaffold tilepath from assemblies obtained at multiple k-mer sizes. Conserved amino acid domains were also used to search for new contigs when scaffolds could not be extended. Gene annotation was carried out manually. Operations were carried out using Geneious V6 (Biomatters Ltd). Gene models were submitted to NCBI with accession numbers listed in Table S1.

### Molecular evolution analysis

For phylogenetic analysis, translated sequences were cropped to either the homeodomain only or the homeodomain plus C-terminal sequence until the deduced stop codon (‘extended’ sequences) (for deduced translations see Figure S5). The extended sequences were aligned using Cobalt [51], and edited to exclude sites with a>50% missing data. Maximum-likelihood trees were built using RAxML [52] with an LG+Γ model and 500 bootstrap replicates. Bayesian analysis was performed using Phylobayes-MPI with a C20 pre-defined mixture of profile and a gamma distribution of among-site rate variation [53].

To evaluate the selective processes at play through the evolution of Shx genes, the d_N_/d_S_ ratio (or ω ratio) of the synonymous and non-synonymous rates of substitution was estimated in a maximum likelihood framework using the codeml program of the PAML package [54]. The ‘branch’ model was employed to evaluate the selective effects along the branches leading to distinct groups of Shx genes (topology as in Figure 1) by assigning 2, 3 or 6 independent ω ratios. Alternatively, site models and branch-site models were employed to assess positive selection at the codon level and the significance of selective effect was assessed using a likelihood ratio test. The probability of sites being under positive selective was evaluated using Bayes Empirical Bayes criteria (posterior probability>0.95).

To search for additional motifs outside the homeodomain, deduced translations of genes from Diptera, Trichoptera and Lepidoptera were aligned using Cobalt. Dipteran analysis used the Mothfly (*Clogmia albipunctata*), Horsefly (*Haematopota pluvialis*), Dancefly (*Empis livida*), Scuttlefly (*Megaselia abdita*), Fruit fly (*Drosophila melanogaster*) and Marmalade Hoverfly (*Episyrphus balteatus*). Lepidopteran analysis used the five genomes sequenced in the current study plus *H. melpomene* and the Diamondback moth *Plutella xylostella*. Caddisfly (Trichoptera) sequences were from the present study, and were compared to the Diptera/Lepidoptera alignments (Figure S5). Conserved motifs were defined as three or more consecutive amino acids present in at least half the species examined and where each residue is shared between divergent lineages (for Lepidoptera one of *Hepialus/Cameraria/Plutella* vs. one of *Heliconius*/*Polygonia*/*Pararge*).

### RT-PCR and in situ hybridisation in Speckled Wood butterfly

We examined the spatial and temporal expression patterns of the 4 *Shx* genes and *zen* in the Speckled Wood (*Pararge aegeria*) using RT-PCR and whole mount *in situ* hybridization (WMISH). Since *zen* is involved in extra-embryonic tissue formation in other winged (pterygote) insects, we paid particular attention to oogenesis and early embryonic development. For RT-PCR analysis, RNA was extracted from eggs and ovaries obtained from mated 4-day old females taken from a large outbred laboratory stock [31]. To examine zygotic expression, fifty embryos were pooled for time points 0, 1, 2, 3, 6, 8, 10, 12, 15, 20, 25, 30 and 48 hours after egg laying (AEL) in triplicate. In Lepidoptera, egg laying is nearly synchronous with fertilization, and time after egg laying (AEL) can therefore be taken as a proxy for time of development. To examine maternal expression, two mated 4-day old females were sacrificed, the abdomens removed and ovaries dissected in ice-cold PBS. Previtellogenic and vitellogenic regions were separated before RNA extraction [31]. RNA was purified using RNeasy Mini Kit (Qiagen) and cDNA synthesized using BioScript (Bioline). The expression of *zen* and Shx genes was assessed by 35 cycles of RT-PCR using GoTaq polymerase (Promega) and primer annealing temperatures of 55°C. All primers are given in Table S4 and their respective position and orientation in Figure S10.

Riboprobes were synthesized using a T7/SP6 DIG RNA labeling Kit (Roche Applied Science, Penzberg, Germany) either from linearized plasmids (*ShxA*, *B*, *zen*) or PCR amplified templates from Speckled Wood cDNA (*ShxC*, *D*) purified using QIAquick (Qiagen, Hilden, Germany). In the latter method, initial amplifications using gene-specific primers were followed by a second PCR implementing a modified reverse or forward primer with a T7 5′ tail (5′-TAATACGACTCACTATAGGG+Fw/Rev-3′) resulting in an antisense or sense template. Regions of genes targeted for RT-PCR and for WMISH are shown in Figure S10. *In-situ* hybridisation was carried out on 10 and 12 h AEL eggs which had been dechorionated prior to fixation using 4% sodium hypochlorite. Ovarioles and embryos were fixed in a 1∶1 mixture of heptane and 5.5% formaldehyde in PBS in glass vials (30 min at 25°C, then 4°C overnight, with gentle rotation) before gradual dehydration in methanol and storage at −20°C. Samples were hybridized with the riboprobes at 55°C and processed as detailed in Note S2, developed from Brakefield et al. (2009) [55]. After WMISH, samples were counter stained with SYTOX Green (Invitrogen; 450–490 nm) and imaged using a MZ FL III Stereo-Fluorescence Microscope (Leica, Wetzlar, Germany) using a ProgResC3 sensor (Jenoptik, Jena, Germany).

### Protein modelling

The program MODELLER-9v7 [56] was used to model deduced Shx and zen homeodomain sequences onto a previously published crystal structure of the *Drosophila* Antp homeodomain bound to the DNA sequence AGAAAGCCATTAGAG (pdb code 9ant; [57]). Energetic stability of sequences was initially assessed using the sum of pairwise atomic interactions, estimated solvent interaction and overall combined energy (see Note S1). To assess stability of binding to the 9ant DNA sequence, the ROSETTA program was deployed (see Note S1) [58]. To identify energetically preferred DNA target sequences for each homeodomain, an *in silico* evolution approach was applied to Shx and zen homeodomains of Speckled Wood, Comma and Horse Chestnut Leafminer. Proteins were modelled in complex with homopolymer sequences using ROSETTA and then random changes introduced over the 11-core positions with elevated sampling in the inner 9 positions. After each round of mutation protein-DNA complexes were remodelled, side-chain and base-pair packing energies recalculated, and the lowest energy structure, as assessed using ROSETTA and dFIRE3, used as the next template for mutation [59]. After 1000 rounds of mutation, starting from each homopolymer run, the DNA sequences associated with the 50 lowest energy structures were used to build consensus sequences. Finally, substitution without *in silico* evolution was used to test for bias introduced from starting with homopolymer runs (see Note S1). Structures were displayed using CHIMERA [60] and consensus DNA sequences with WebLogo (http://weblogo.berkeley.edu).

## Supporting Information

Figure S1 Homeodomain phylogenetic tree. Maximum likelihood tree obtained from homeodomain alignment using RAxML and LG+Γ model. Support values are majority-rule consensus from 200 bootstrap replicates.
(PDF)

Figure S2 (A) Summary of the conserved motifs identified from alignments of the deduced protein sequences (see Figure S5). Green box, motif; blue box, homeodomain; dashed lines join motifs shared between genes. All proteins are drawn to scale, and homeodomains aligned. A/B-box - fly lineage motifs identified by Stauber *et al.* [12]. The lepidopteran zen highly conserved ‘YSP’ and ‘PNG’ motifs are starred. The region N-terminal of the homeobox in ShxB and D could be annotated with confidence in only two species, motifs have therefore not been defined in this region. (B) Heatmap representation of pairwise divergence between full-length Shx and zen proteins assuming a ML distance (JTT model). The species order is indicated.
(EPS)

Figure S3 Extended alignment phylogenetic tree. Maximum likelihood tree obtained from an extended alignment encompassing conserved protein domains outside the homeodomain. The tree was inferred using RAxML and a LG+Γ model. Support values are majority-rule consensus from 200 bootstrap replicates.
(PDF)

Figure S4 Phylogenetic tree including *Bombyx*. Bayesian tree obtained from a homeodomain alignment incorporating Shx and Hox genes from a broader set of species including *Bombyx mori* that was excluded from primary analysis for clarity. The tree was inferred using Phylobayes assuming a C20 mixure of profiles and support values are posterior probabilities.
(PDF)

Figure S5 Full length deduced protein alignments. The homeodomain is boxed in red; conserved motifs illustrated in Figure S2 are shaded in green. Divergent amino acids are highlighted. (A) pb, (B) Dfd, (C–F) ShxA-D (G) zen, motifs shared between the caddisfly and lepidoptera or flies are shaded in blue and orange respectively. The highly conserved YSP and PNG motifs are starred. (H) Fly zen with conserved regions A/B-box identified by Stauber *et al.* [39] highlighted in orange. C-H- Conserved motifs are defined by the consensus sequence, which was adjusted according to the rules laid out in the Methods section. (I) Consensus homeodomains extracted from C-H with significant residues indicated by red and green boxes (see main text).
(PDF)

Figure S6 Detection of positive selection. Tree showing dN (coding substitutions) as branch length and ω (dN/dS) ratios as branch label, inferred by PAML. The putative episode of positive selection in the lineage leading to ShxB/C/D is highlighted.
(PDF)

Figure S7 Additional observations of Shx expression. Production and subsequent localisation of *ShxC* transcripts as shown in early (A), mid (B) and late stage (C) *P. aegeria* follicles. *ShxC* and *ShxD* localisation in early *P. aegeria* embryos (E to I). *ShxC* maternal transcript ‘hourglass’ distribution in the embryo cortex as blastoderm cellularisation begins (approx. 8 h) (D and G). *ShxD* expression in 10 h embryos (E, F, H and I). *ShxA* expression in median sagittal section through 12 h blastoderm and yolk cells (J). *Pa-zen* expression in 12 h embryo (K). Sytox Green staining in 8 h (L), 10 h (M), 11 h (N) and 12 h (O–P) embryos (see Figure 3B for schematic representation); panels L, M and P are complementary to G, H and K. Oocytes mature in sequence, with the more mature oocytes on the right and the germarium on the bottom left in the composite (A–C) with ventral (B) and lateral (C) facing oocytes. Embryos are oriented to show anterior pole (D–F), ventral (H, K, M–P), dorsal (I) and ventro-lateral faces (G, L, O). Red arrows indicate anterior pole (D–F) and blastoderm/yolk cell boundary (J). Green arrows indicate first signs of anterior blastoderm cell formation (D, G) as cleavage nuclei reach periplasm (L). Orientation for each panel is indicated in bottom right 3D axis indicating anterior (A) or posterior (P), left (L) or right (R) and ventral (V) or dorsal (D). All time-points are AEL (After egg-laying). Scale bars 200 µm.
(TIF)

Figure S8 Overview of embryonic tissue movements following serosal closure. Schematic recapitulating serosal closure (**A**, **B** and **C**) and the distinctive embryonic tissue movements that follow (**D**, **E** and **F**) in butterflies. The initially wide germ anlage converges to the ventral side while head lobes begin to take form (‘pyriform stage’, **E**). The germ band will continue to contract and elongate to reach a ‘spoon-shaped’ stage (**F**) at which point gastrulation begins. Segmentation will then occur from anterior to posterior. Tissues are pseudo-translucent with embryonic edges on opposing side represented in dotted lines. Arrows indicate ongoing movements/contractions. Orientation 3D axis indicates anterior (A), left (L) and dorsal (D) or ventral (V), the top row shows the dorsal face while the bottom row shows the ventral face.
(TIF)

Figure S9 K-mer spectrum in the lepidopteran and trichopteran sequences obtained in this study. The number of 17-mers represented at a given coverage is plotted as a histogram; low frequency k-mers correspond to sequencing errors introducing random mutations while the repeated elements of the genome are responsible for high frequency k-mers. The peak indicates the k-mer coverage (red line) related to sequencing depth.
(PDF)

Figure S10 Overview of primer binding sites for RT-PCR and hybridization targets.
(TIF)

Table S1 Gene model accession numbers.
(DOCX)

Table S2 Results of PAML selection analysis for M1-M7 site models and for the branch-site model. For each residue of the homeodomain, the probability of belonging to a given category of ω ratio as well as the inferred ω ratio is included. H1, H2, H3 denote residues in alpha helices. Sites with evidence of unconstrained evolution or positive selection are marked with asterisks.
(XLSX)

Table S3 Source of samples. Taxonomy assignment and geographic origin of individuals used for genome sequencing.
(DOCX)

Table S4 Primer sequences and properties. Forward and reverse primer sequences used for RT-PCR analysis and for primary and secondary riboprobe template generation; annealing temperatures in degrees Celsius (Ta) and amplicon size in base pairs (bp) for each pairing.
(DOCX)

Note S1 Methods used for molecular modelling of homeodomains.
(PDF)

Note S2 Whole Mount *in situ* Hybridisation on *Pararge aegeria* ovarioles and embryos.
(DOCX)
