# Supplementary figures and images for: Ancient Expansion of the Hox Cluster in Lepidoptera Generated Four Homeobox Genes Implicated in Extra-Embryonic Tissue Formation
Source: PLoS Genet. 2014 Oct 23;10(10):e1004698. doi: 10.1371/journal.pgen.1004698 (PMC4207634; doi:10.1371/journal.pgen.1004698)

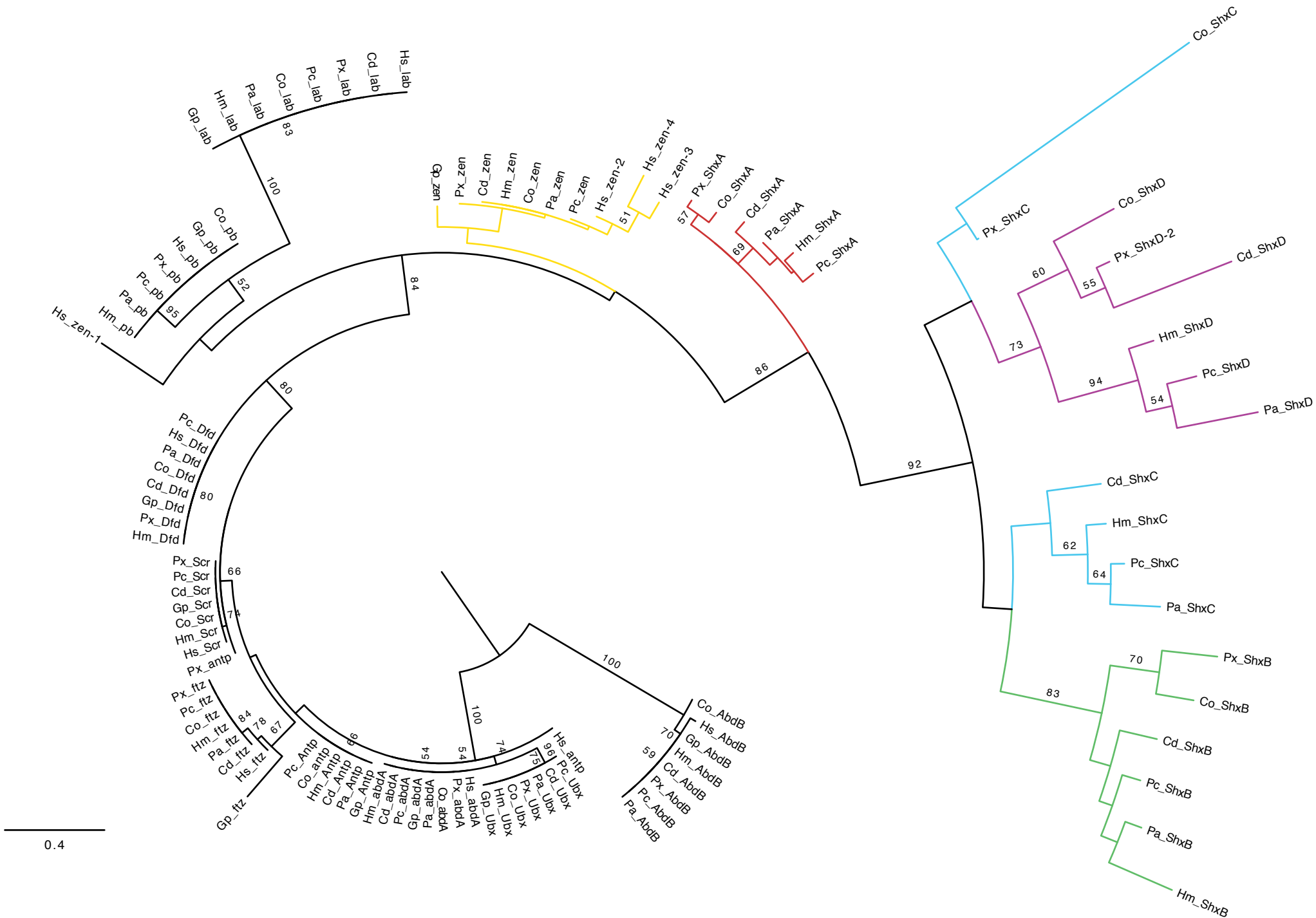

Supplement: Figure S1 — Homeodomain phylogenetic tree. Maximum likelihood tree obtained from homeodomain alignment using RAxML and LG+Γ model. Support values are majority-rule consensus from 200 bootstrap replicates. (PDF) [file pgen.1004698.s001.pdf]

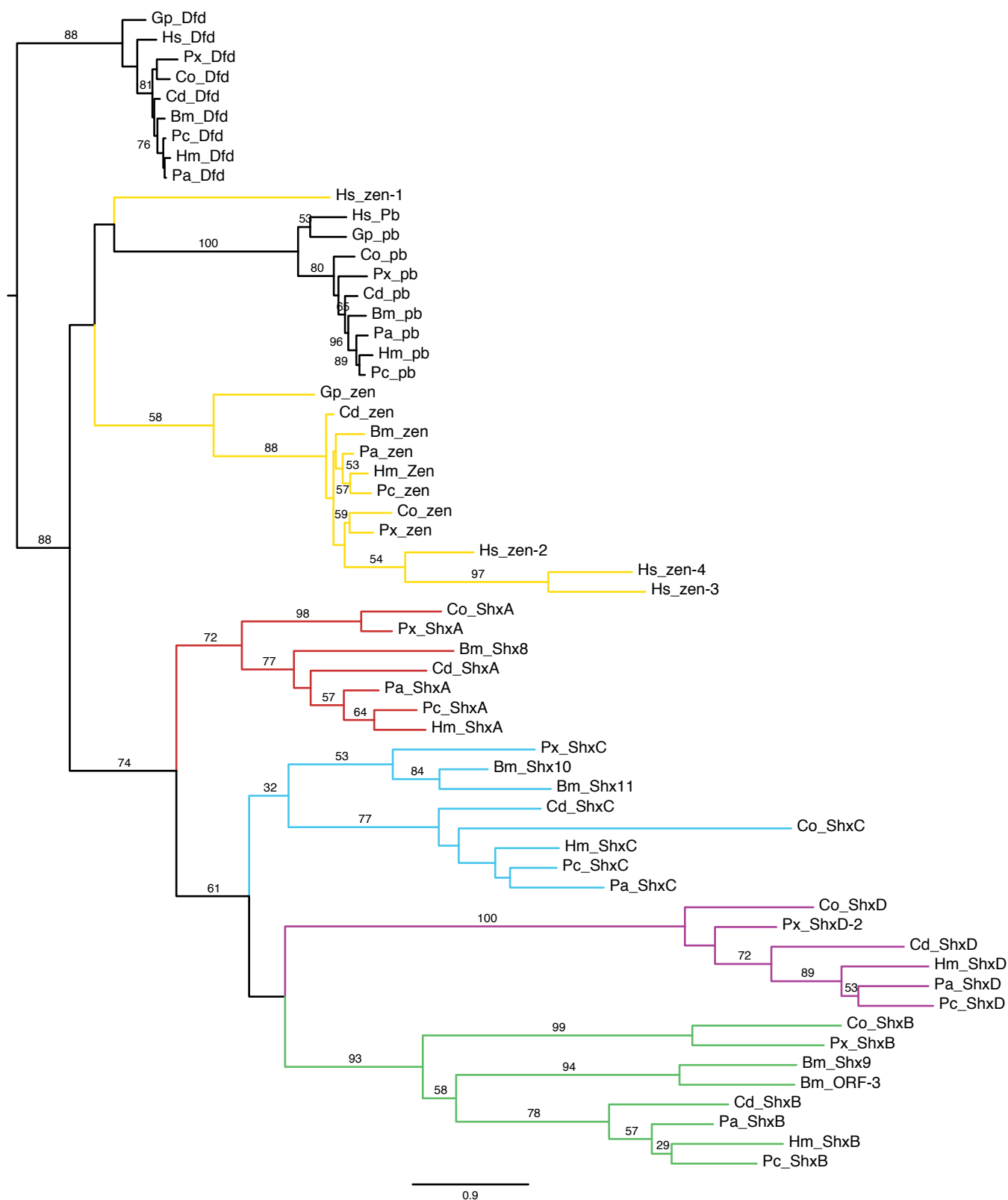

Supplement: Figure S3 — Extended alignment phylogenetic tree. Maximum likelihood tree obtained from an extended alignment encompassing conserved protein domains outside the homeodomain. The tree was inferred using RAxML and a LG+Γ model. Support values are majority-rule consensus from 200 bootstrap replicates. (PDF) [file pgen.1004698.s003.pdf]

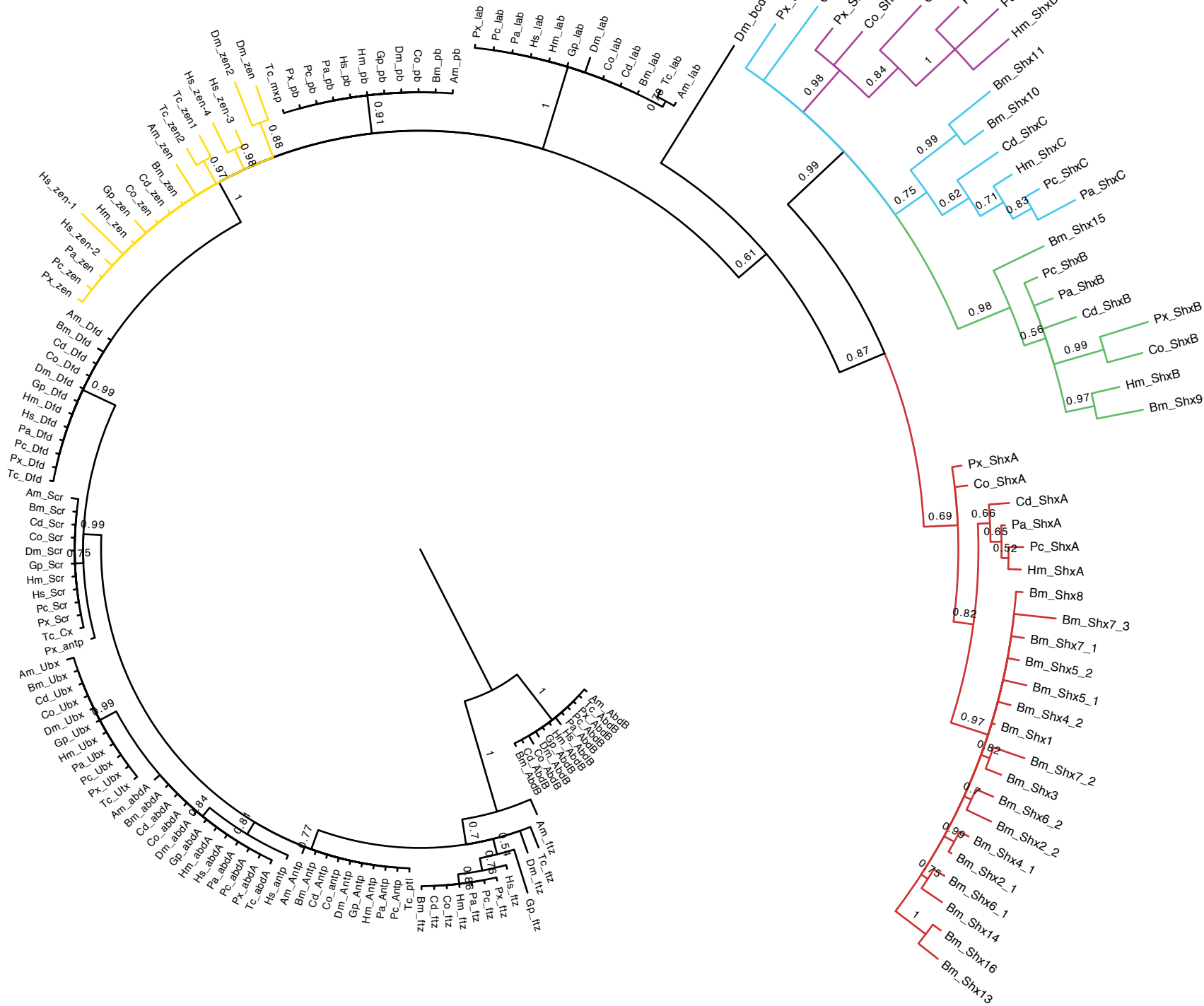

Supplement: Figure S4 — Phylogenetic tree including Bombyx. Bayesian tree obtained from a homeodomain alignment incorporating Shx and Hox genes from a broader set of species including Bombyx mori that was excluded from primary analysis for clarity. The tree was inferred using Phylobayes assuming a C20 mixure of profiles and support values are posterior probabilities. (PDF) [file pgen.1004698.s004.pdf]

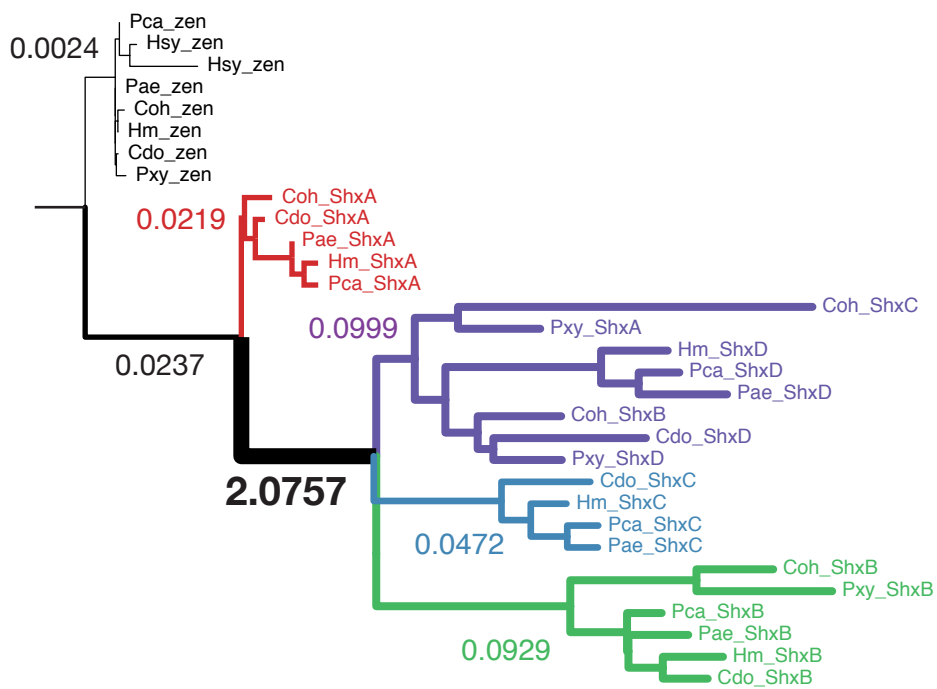

Supplement: Figure S6 — Detection of positive selection. Tree showing dN (coding substitutions) as branch length and ω (dN/dS) ratios as branch label, inferred by PAML. The putative episode of positive selection in the lineage leading to ShxB/C/D is highlighted. (PDF) [file pgen.1004698.s006.pdf]

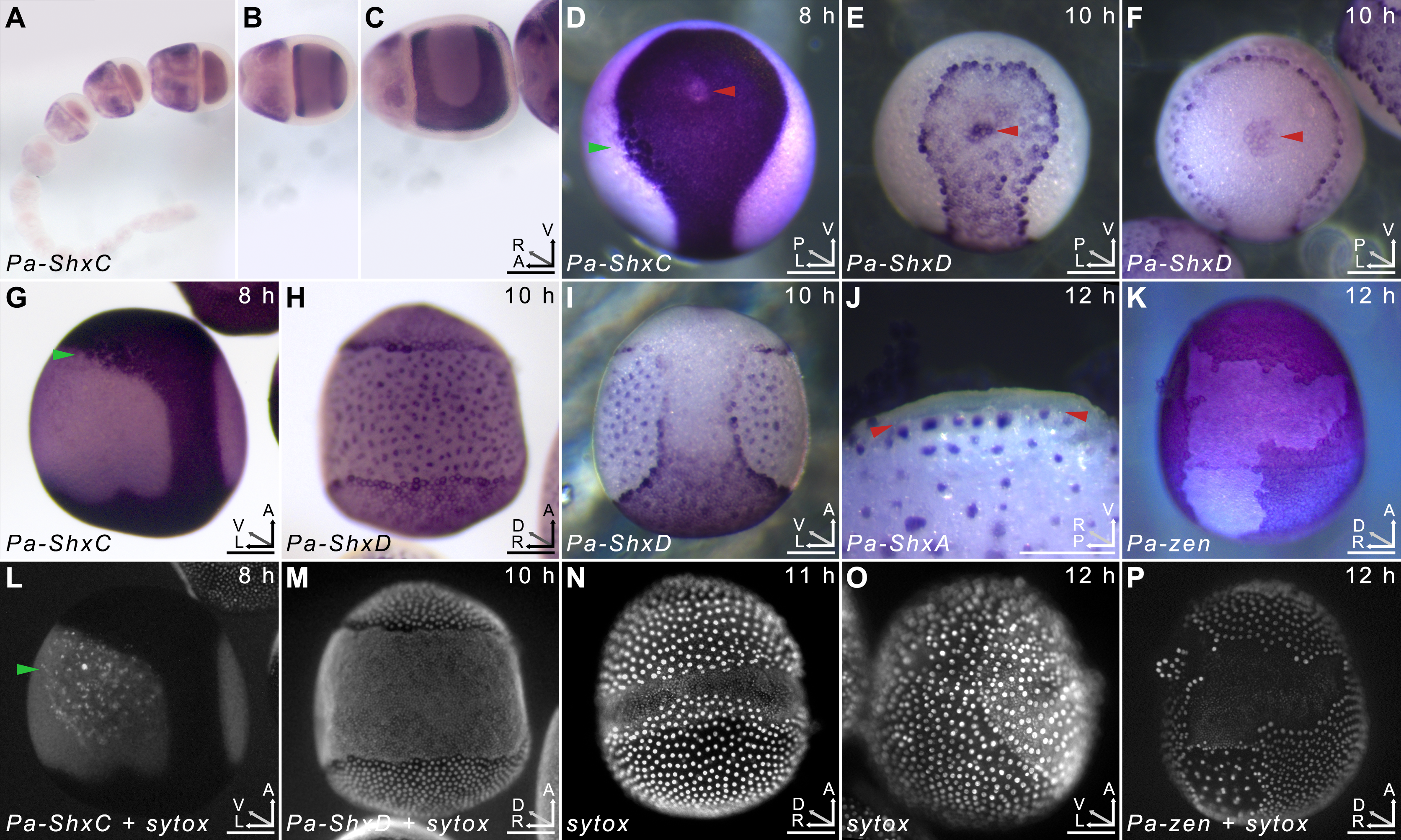

Supplement: Figure S7 — Additional observations of Shx expression. Production and subsequent localisation of ShxC transcripts as shown in early (A), mid (B) and late stage (C) P. aegeria follicles. ShxC and ShxD localisation in early P. aegeria embryos (E to I). ShxC maternal transcript ‘hourglass’ distribution in the embryo cortex as blastoderm cellularisation begins (approx. 8 h) (D and G). ShxD expression in 10 h embryos (E, F, H and I). ShxA expression in median sagittal section through 12 h blastoderm and yolk cells (J). Pa-zen expression in 12 h embryo (K). Sytox Green staining in 8 h (L), 10 h (M), 11 h (N) and 12 h (O–P) embryos (see Figure 3B for schematic representation); panels L, M and P are complementary to G, H and K. Oocytes mature in sequence, with the more mature oocytes on the right and the germarium on the bottom left in the composite (A–C) with ventral (B) and lateral (C) facing oocytes. Embryos are oriented to show anterior pole (D–F), ventral (H, K, M–P), dorsal (I) and ventro-lateral faces (G, L, O). Red arrows indicate anterior pole (D–F) and blastoderm/yolk cell boundary (J). Green arrows indicate first signs of anterior blastoderm cell formation (D, G) as cleavage nuclei reach periplasm (L). Orientation for each panel is indicated in bottom right 3D axis indicating anterior (A) or posterior (P), left (L) or right (R) and ventral (V) or dorsal (D). All time-points are AEL (After egg-laying). Scale bars 200 µm. (TIF) [file pgen.1004698.s007.tif]

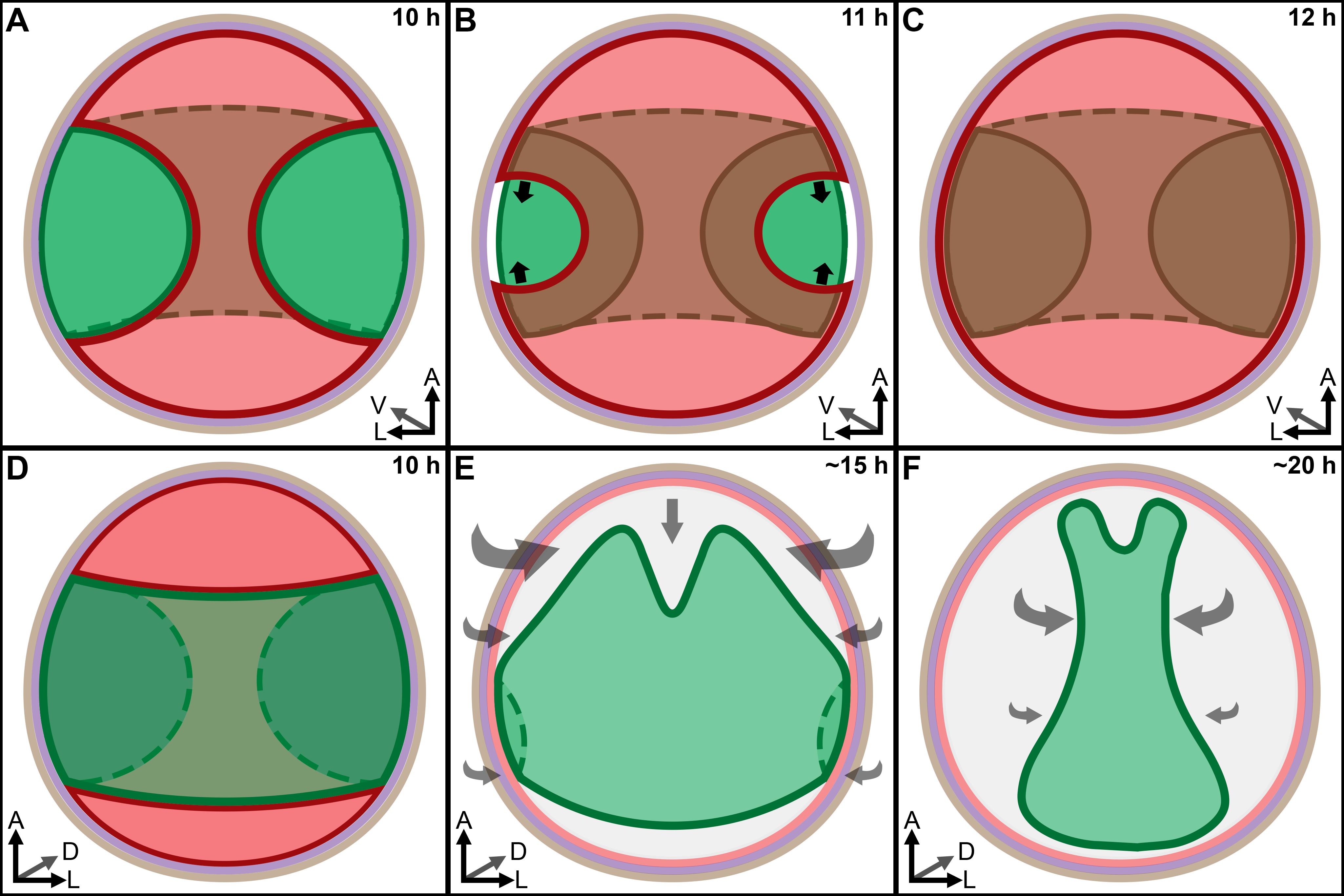

Supplement: Figure S8 — Overview of embryonic tissue movements following serosal closure. Schematic recapitulating serosal closure (A, B and C) and the distinctive embryonic tissue movements that follow (D, E and F) in butterflies. The initially wide germ anlage converges to the ventral side while head lobes begin to take form (‘pyriform stage’, E). The germ band will continue to contract and elongate to reach a ‘spoon-shaped’ stage (F) at which point gastrulation begins. Segmentation will then occur from anterior to posterior. Tissues are pseudo-translucent with embryonic edges on opposing side represented in dotted lines. Arrows indicate ongoing movements/contractions. Orientation 3D axis indicates anterior (A), left (L) and dorsal (D) or ventral (V), the top row shows the dorsal face while the bottom row shows the ventral face. (TIF) [file pgen.1004698.s008.tif]

**Speckled wood butterfly**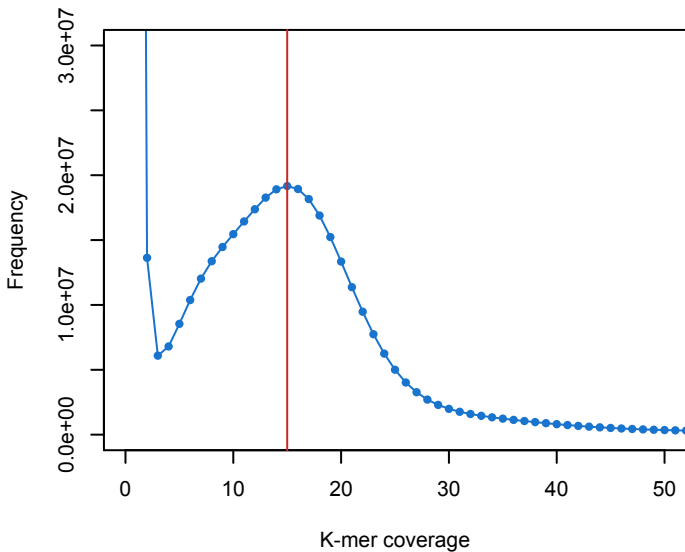**Comma butterfly**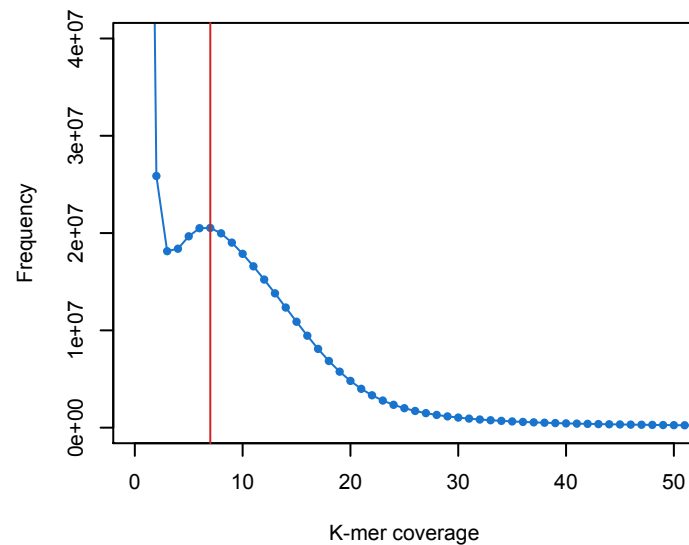**Tiger moth**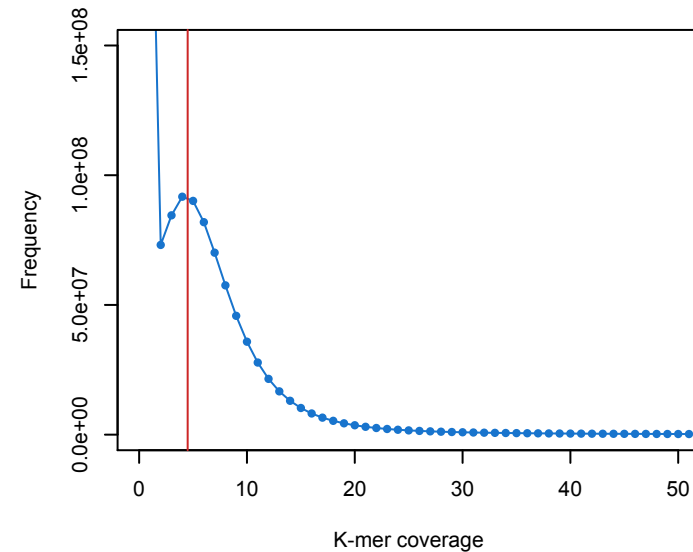**Chesnut moth**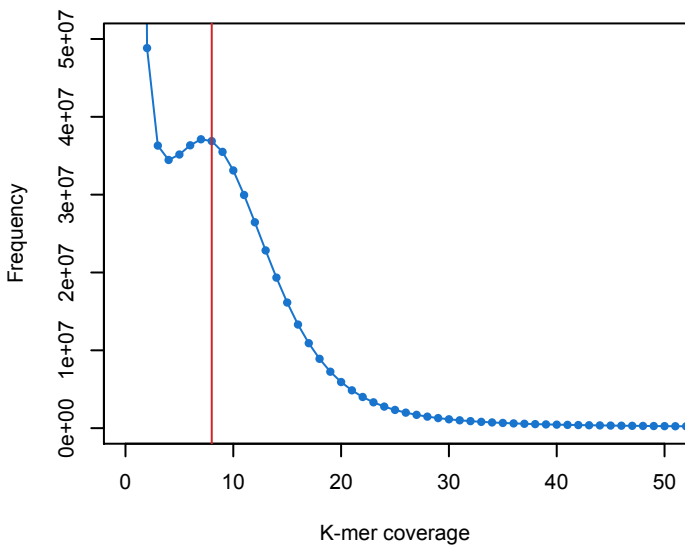**Swift moth**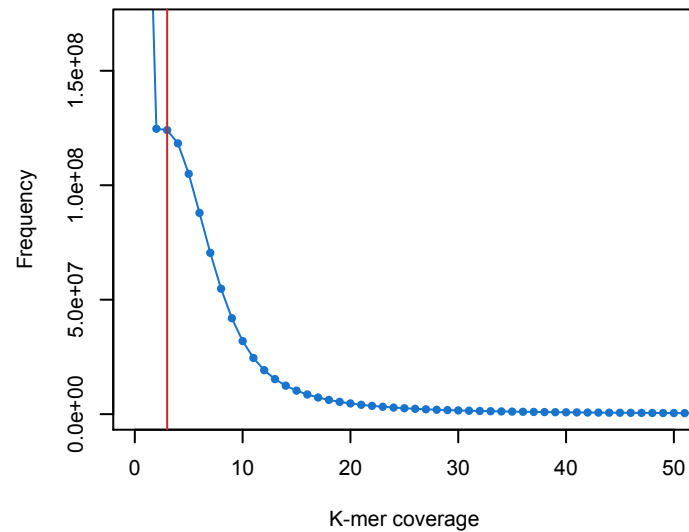**Caddisfly**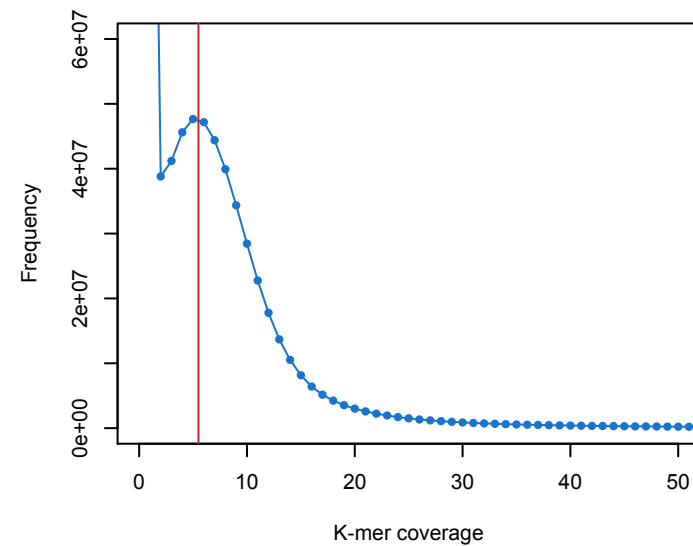

Supplement: Figure S9 — K-mer spectrum in the lepidopteran and trichopteran sequences obtained in this study. The number of 17-mers represented at a given coverage is plotted as a histogram; low frequency k-mers correspond to sequencing errors introducing random mutations while the repeated elements of the genome are responsible for high frequency k-mers. The peak indicates the k-mer coverage (red line) related to sequencing depth. (PDF) [file pgen.1004698.s009.pdf]

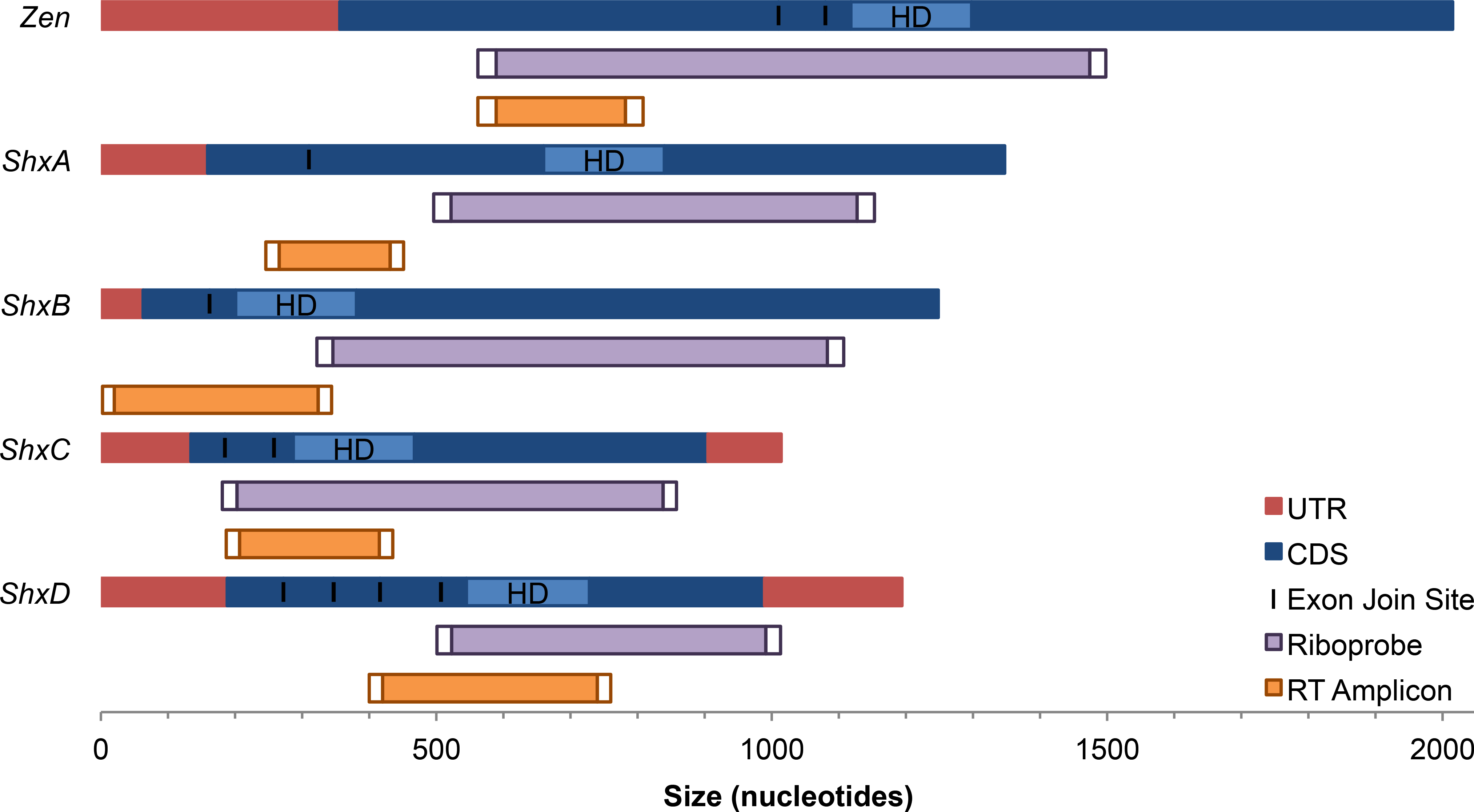

Supplement: Figure S10 — Overview of primer binding sites for RT-PCR and hybridization targets. (TIF) [file pgen.1004698.s010.tif]
