# Supplementary material for: Ancient Expansion of the Hox Cluster in Lepidoptera Generated Four Homeobox Genes Implicated in Extra-Embryonic Tissue Formation
Source: PLoS Genet. 2014 Oct 23;10(10):e1004698. doi: 10.1371/journal.pgen.1004698 (PMC4207634; doi:10.1371/journal.pgen.1004698)

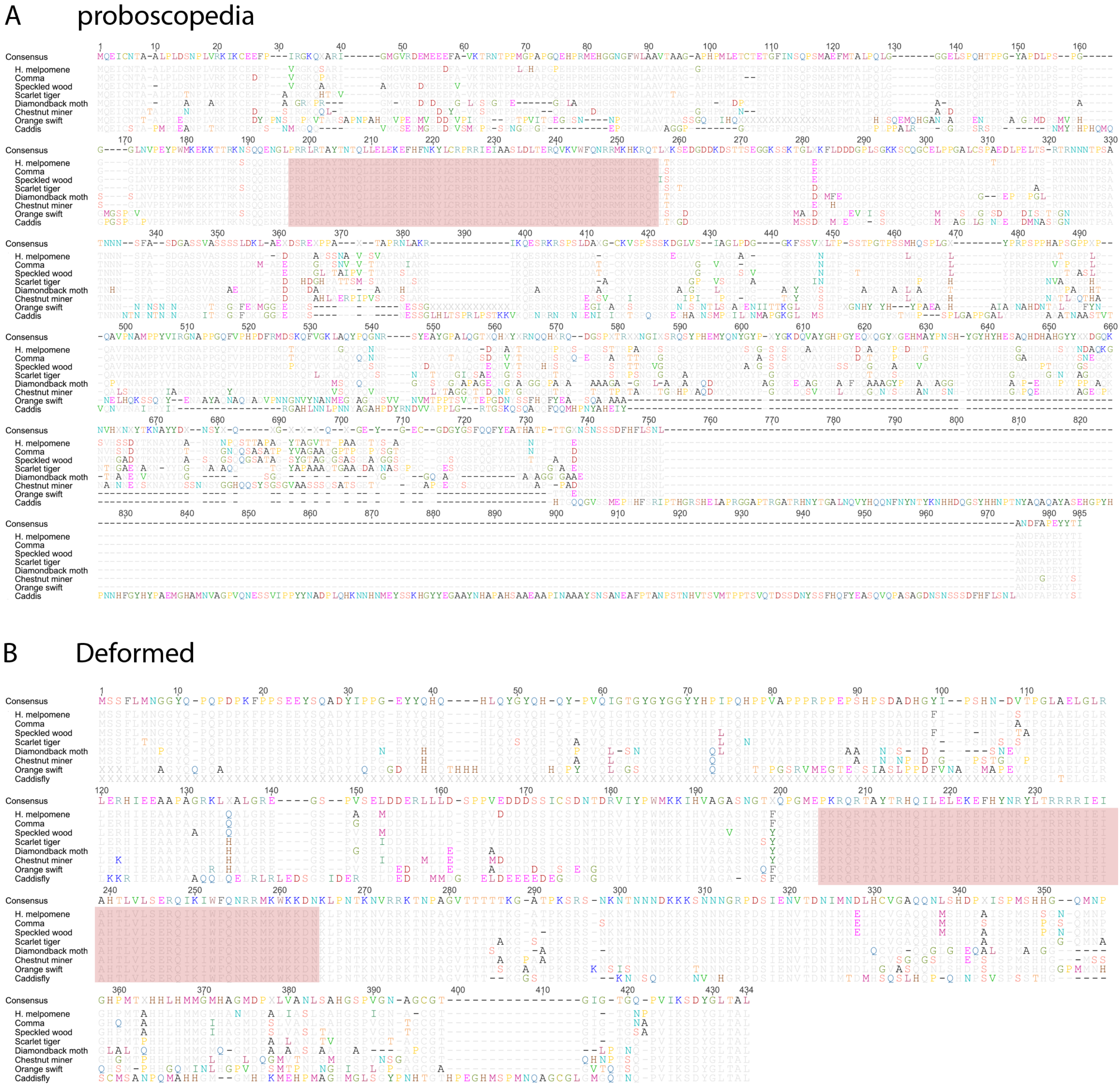
A
proboscopedia
B
Deformed
Consensus
H. melpomene
Comma
Speckled wood
Scarlet tiger
Diamondback moth
Chestnut miner
Orange swift
Caddis
Caddisfly

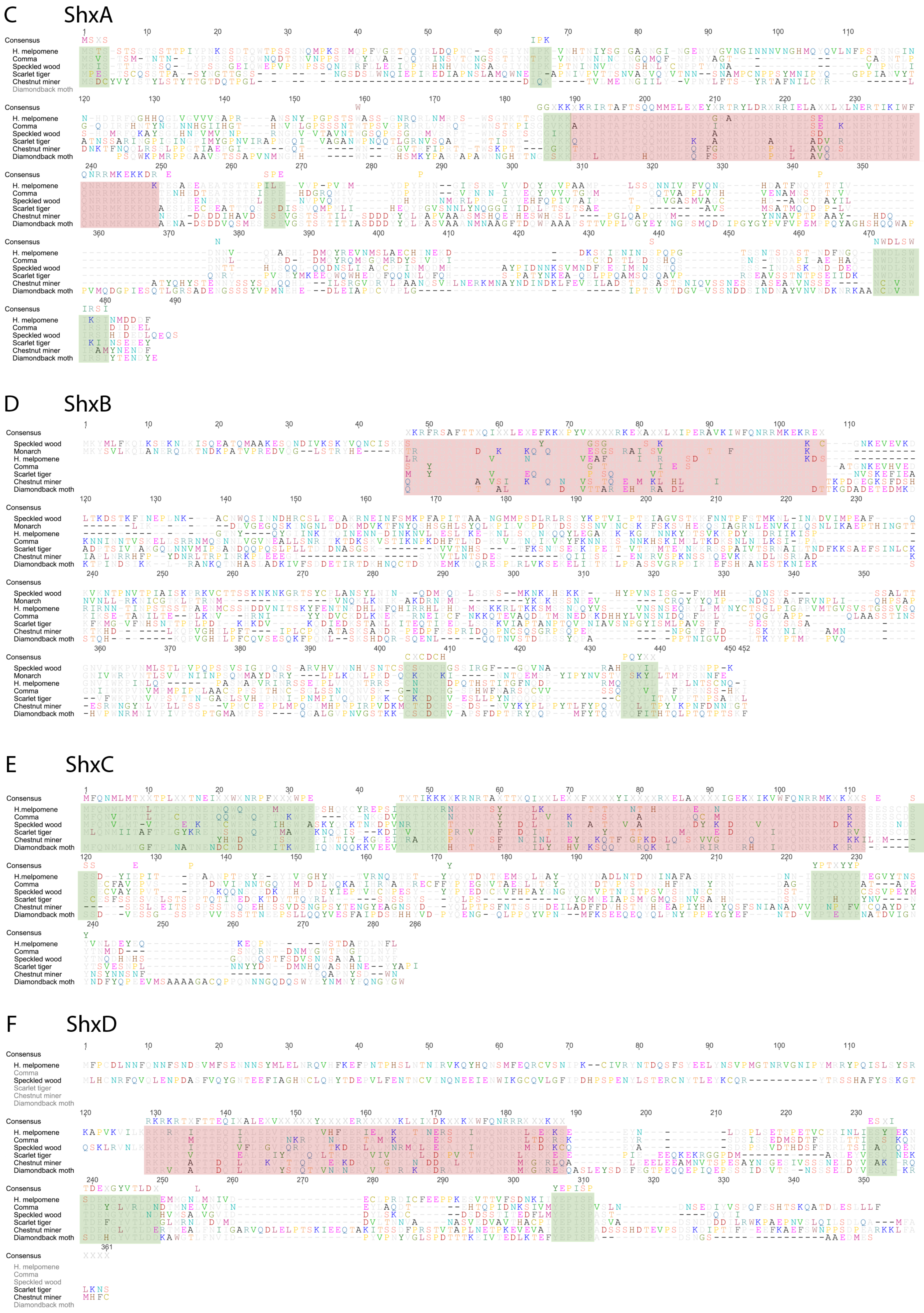
C
ShxA
D
ShxB
E
ShxC
F
ShxD
Consensus
H. melpomene
Comma
Speckled wood
Scarlet tiger
Chestnut miner
Diamondback moth
Monarch

G Lepidopteran and caddisfly zen alignment

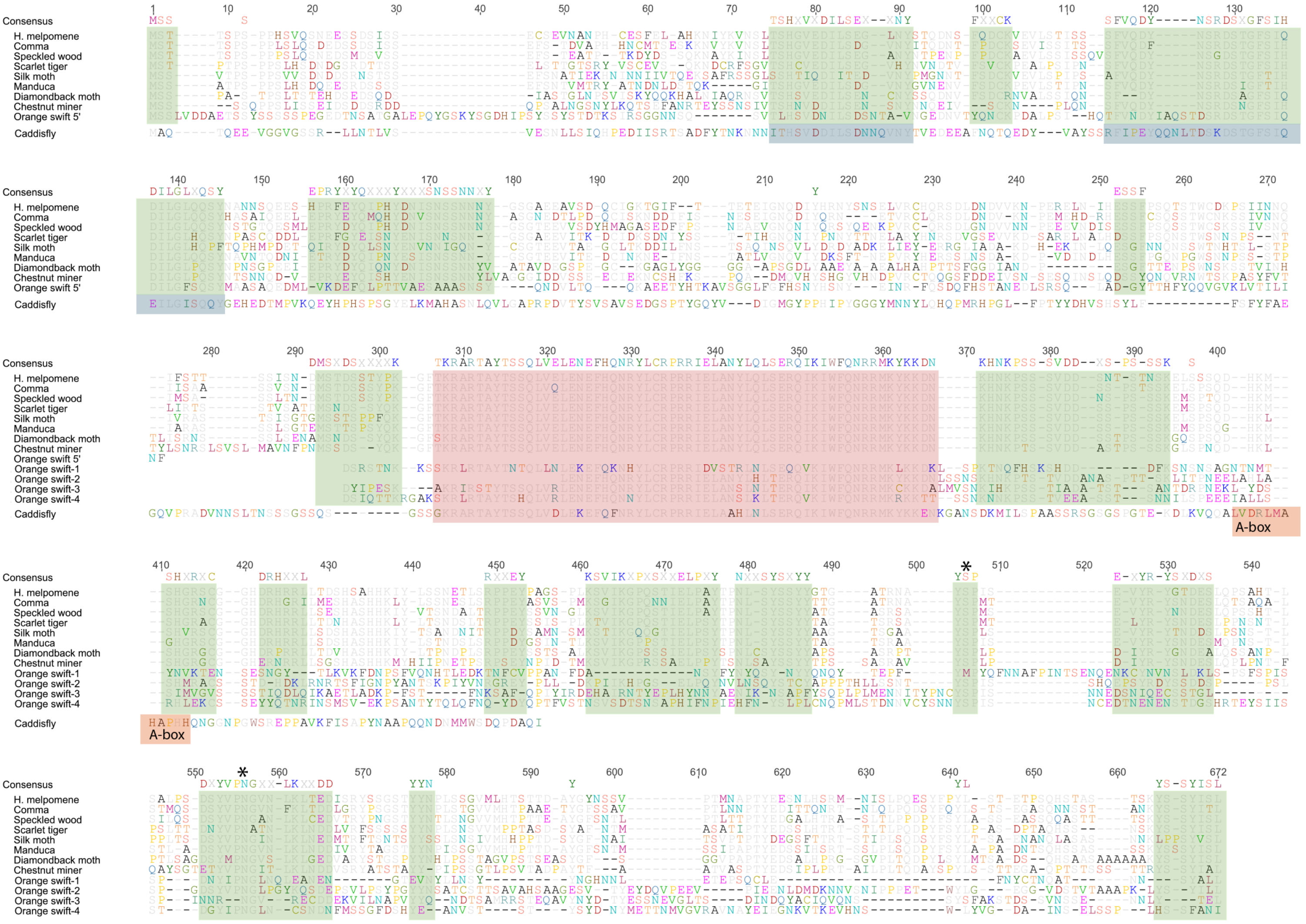

H Fly zen alignment

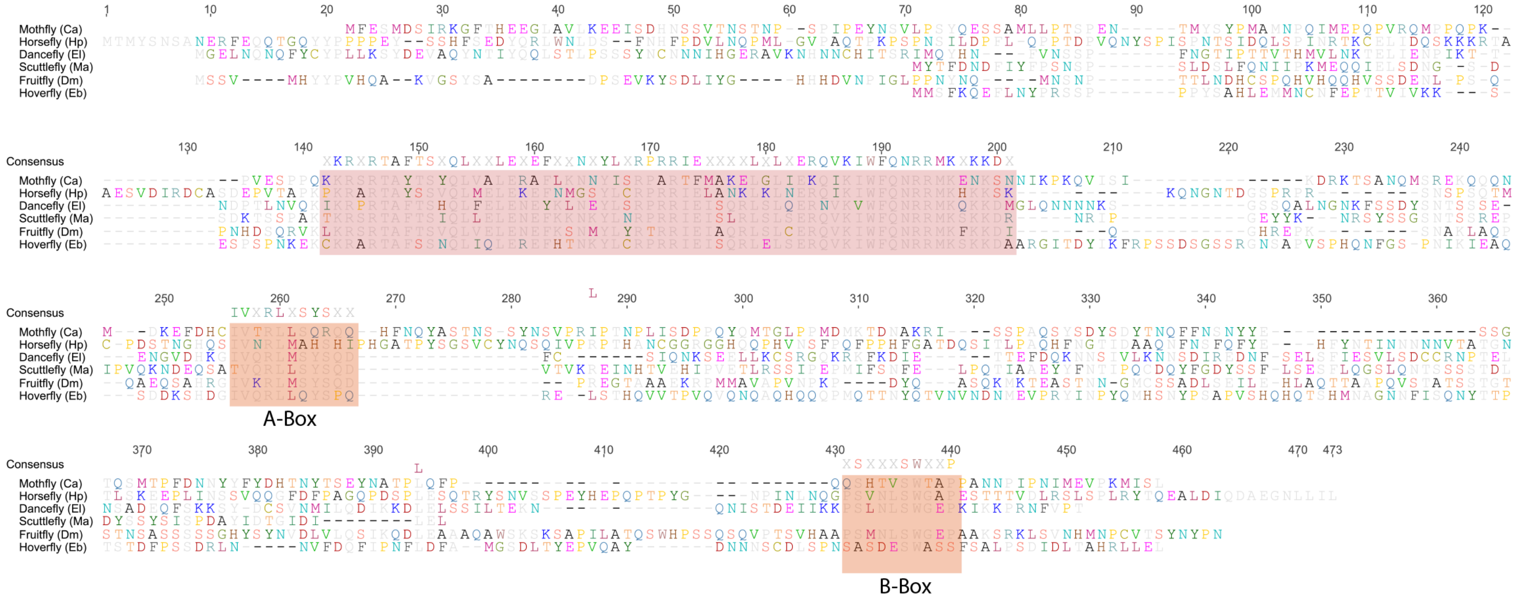

I Zen and Shx homeodomain alignment

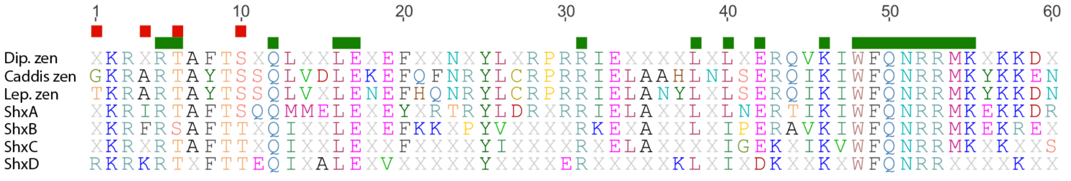

Supplement: Figure S5 — Full length deduced protein alignments. The homeodomain is boxed in red; conserved motifs illustrated in Figure S2 are shaded in green. Divergent amino acids are highlighted. (A) pb, (B) Dfd, (C–F) ShxA-D (G) zen, motifs shared between the caddisfly and lepidoptera or flies are shaded in blue and orange respectively. The highly conserved YSP and PNG motifs are starred. (H) Fly zen with conserved regions A/B-box identified by Stauber et al. [39] highlighted in orange. C-H- Conserved motifs are defined by the consensus sequence, which was adjusted according to the rules laid out in the Methods section. (I) Consensus homeodomains extracted from C-H with significant residues indicated by red and green boxes (see main text). (PDF) [file pgen.1004698.s005.pdf]
