# Supplementary material for: Ancient Expansion of the Hox Cluster in Lepidoptera Generated Four Homeobox Genes Implicated in Extra-Embryonic Tissue Formation
Source: PLoS Genet. 2014 Oct 23;10(10):e1004698. doi: 10.1371/journal.pgen.1004698 (PMC4207634; doi:10.1371/journal.pgen.1004698)
Supplement: Table S1 — Gene model accession numbers. (DOCX) [file pgen.1004698.s011.docx]

| Species | Scaffold containing gene | GenBank accession number |
| --- | --- | --- |
| Scarlet Tiger moth | ShxA | KJ739621 |
|  | ShxB | KJ739618 |
|  | ShxC, ShxD | KJ739620 |
|  | zen | KJ739619 |
|  | pb | KJ739625 |
|  | Dfd | KJ739624 |
| Horse Chestnut Leafminer moth | ShxA | KJ739622 |
|  | ShxB, ShxC | KJ739628 |
|  | ShxD | KJ739629 |
|  | zen | KJ739623 |
|  | pb | KJ739627 |
|  | Dfd | KJ739626 |
| Caddisfly | zen | KJ739632 |
|  | pb | KJ739631 |
|  | Dfd | KJ739630 |
| Orange Swift moth | Zen-1 | KJ739635 |
|  | Zen-2 | KJ739636 |
|  | Zen-3 | KJ739637 |
|  | Zen-4 | KJ739638 |
|  | pb | KJ739634 |
|  | Dfd | KJ739633 |
| Speckled Wood butterfly | ShxA, ShxB | KJ739640 |
|  | ShxC, ShxD, zen | KJ739641 |
|  | pb | KJ739639 |
|  | Dfd | KJ739642 |
| Comma butterfly | ShxA, ShxB, ShxC, ShxD, zen | KJ739644 |
|  | pb | KJ739643 |
|  | Dfd | KJ739645 |
|  |  |  |
| Third Party Annotation gene  Open Reading Frame only: |  |  |
| Diamondback moth | ShxB | BK008849 |
|  | ShxC | BK008850 |
|  | ShxD | BK008851 |
|  | zen | BK008852 |
| Domesticated Silkmoth | Shx9 (ShxB) | BK008855 |
|  | Shx10 (ShxC-1) | BK008856 |
|  | Shx11 (ShxC-2) | BK008857 |
|  | Shx13 | 57bp ORF positions  2,324,407-2,324,463  Bm_scaf11\|DF090326 |
|  | Shx14 | BK008858 |
|  | Shx15 | BK008859 |
|  | Shx16 | 177bp ORF positions  925-1101  Bm_scaf1030\|BABH01045148 |
