# Supplementary material for: Ancient Expansion of the Hox Cluster in Lepidoptera Generated Four Homeobox Genes Implicated in Extra-Embryonic Tissue Formation
Source: PLoS Genet. 2014 Oct 23;10(10):e1004698. doi: 10.1371/journal.pgen.1004698 (PMC4207634; doi:10.1371/journal.pgen.1004698)
Supplement: Table S3 — Source of samples. Taxonomy assignment and geographic origin of individuals used for genome sequencing. (DOCX) [file pgen.1004698.s013.docx]

| **Common Name** | **Species Name** | **Order** | **Family** | **Number of individuals** | **Location** | **UK grid reference** | **Date** |
| --- | --- | --- | --- | --- | --- | --- | --- |
| Caddisfly | *Glyphotaelius pellucidus* | Trichoptera | Limnephilidae | Single adult | Oxford, UK | SP526068 | 11/08/2011 |
| Orange Swift moth | *Hepialus sylvina* | Lepidoptera | Hepialidae | Single adult | Wallingford, Oxfordshire, UK | SU597899 | 21/08/2011 |
| Horse-Chestnut  Leaf miner moth | *Cameraria ohridella* | Lepidoptera | Gracillariidae | 75 adults | Reared from Horse Chestnut leaves, Wallingford, Oxfordshire (single tree) | SU619884 | 21/07/2012 |
| Scarlet Tiger moth | *Callimorpha dominula* | Lepidoptera | Arctiidae | Single adult | Wallingford, Oxfordshire, UK | SU597899 | 09/06/2012 |
| Speckled Wood butterfly | *Pararge aegeria* | Lepidoptera | Nymphalidae | Single adult | Belgium Stock: Brookes University, Oxford, UK | n/a | Stock 2007 |
| Comma butterfly | *Polygonia c-album* | Lepidoptera | Nymphalidae | Single adult | UK Stock: Worldwide Butterflies Ltd, UK | n/a | 2010 |

**Table S3: Taxonomic assignment and geographical origin of individuals used for genome sequencing**
