# Supplementary material for: Ancient Expansion of the Hox Cluster in Lepidoptera Generated Four Homeobox Genes Implicated in Extra-Embryonic Tissue Formation
Source: PLoS Genet. 2014 Oct 23;10(10):e1004698. doi: 10.1371/journal.pgen.1004698 (PMC4207634; doi:10.1371/journal.pgen.1004698)
Supplement: Table S4 — Primer sequences and properties. Forward and reverse primer sequences used for RT-PCR analysis and for primary and secondary riboprobe template generation; annealing temperatures in degrees Celsius (Ta) and amplicon size in base pairs (bp) for each pairing. (DOCX) [file pgen.1004698.s014.docx]

| Purpose | Target | Forward Primer Sequence (5'-3') | Reverse Primer Sequence (5'-3') | Ta | Amplicon |
| --- | --- | --- | --- | --- | --- |
| Riboprobe Template | *Pa-ShxA* | AGGCATACAGTGGTCTCCACAATCCA | GCATGTTTATTGGAGGACACTGCGCT | 55.0°C | 657 bp |
| Riboprobe Template | *Pa-ShxB* | CGGAGCGAGCCGTAAAAATTTGGT | ATGAACCCGTGCACTATTTTGGGG | 55.0°C | 785 bp |
| Riboprobe Template | *Pa-ShxC* | AGAGATTAATTGCTGGGAAAAC | CGGAAAATGTGCTTTGTTGA | 65.0°C | 677 bp |
| Riboprobe Template | *Pa-ShxD* | ATTCAAGTAAAGGGACACAAAG | TTATTTGCCTCTATTGTCATGG | 65.0°C | 512 bp |
| Riboprobe Template | *Pa-zen* | TCCGTTTCCAGATTGCAAAGCAGAAGT | TCAGGTGGTCGTGTAAGTGCATCA | 55.0°C | 936 bp |
| RT-PCR | *Pa-ShxA* | CCTAGCTCGCAAAACGAAAC | CATTGACATAGGTGGGAGGG | 55.0°C | 205 bp |
| RT-PCR | *Pa-ShxB* | TCTTCTGATTGTGCTCCGTG | CAAATTTTTACGGCTCGCTC | 55.0°C | 344 bp |
| RT-PCR | *Pa-ShxC* | TAATTGCTGGGAAAACCGAC | GAAACCAAACCTTGACGCAT | 55.0°C | 248 bp |
| RT-PCR | *Pa-ShxD* | AGATCATCCATCACCGGAAA | TCAACTGATGATGGCGAATC | 55.0°C | 360 bp |
| RT-PCR | *Pa-zen* | TCCGTTTCCAGATTGCAAAGCAGAAGT | ACCTCTTCAGTGCCTGATCCGTAGTT | 55.0°C | 246 bp |
| T7 Attachment (-) | *Pa-ShxC* | AGAGATTAATTGCTGGGAAAAC | TAATACGACTCACTATAGGGCGGAAAATGTGCTTTGTTGA | 65.0°C | 697 bp |
| T7 Attachment (-) | *Pa-ShxD* | ATTCAAGTAAAGGGACACAAAG | TAATACGACTCACTATAGGGTTATTTGCCTCTATTGTCATGG | 65.0°C | 532 bp |
| T7 Attachment (+) | *Pa-ShxC* | TAATACGACTCACTATAGGGAGAGATTAATTGCTGGGAAAAC | CGGAAAATGTGCTTTGTTGA | 65.0°C | 697 bp |
| T7 Attachment (+) | *Pa-ShxD* | TAATACGACTCACTATAGGGATTCAAGTAAAGGGACACAAAG | TTATTTGCCTCTATTGTCATGG | 65.0°C | 532 bp |
