# Supplementary material for: Ancient Expansion of the Hox Cluster in Lepidoptera Generated Four Homeobox Genes Implicated in Extra-Embryonic Tissue Formation
Source: PLoS Genet. 2014 Oct 23;10(10):e1004698. doi: 10.1371/journal.pgen.1004698 (PMC4207634; doi:10.1371/journal.pgen.1004698)
Supplement: Note S1 — Methods used for molecular modelling of homeodomains. (PDF) [file pgen.1004698.s015.pdf]

SUPPLEMENTARY MATERIAL

Molecular Modelling Methods for “Ancient expansion of the Hox cluster in Lepidoptera generated four homeobox genes implicated in egg development”

1 Introduction

The methods described in this section were devised to answer the following questions: given a sequence can it be determined if it; 1) is a homeodomain, 2) is structurally stable, 3) has potential to bind to DNA, and 4) has potential to bind to a preferred sequence. We initially applied the method to the five sequences ShxA, ShxB, ShxC, ShxD and zen from the Comma butterfly (*Polygonia c-album*) and then to the same set of sequences in the closely related Speckled Wood butterfly (*Pararge aegeria*) (described in the main text) and also to the more distantly related Horse Chestnut Leafminer moth (*Cameraria ohridella*), the results for which are included below along with those of Comma.

2 Sequence similarity

Based on sequence alignment and phylogenetics, all five sequences show clear similarity with other homeodomain genes and have amino acids at conserved sites that are consistent with DNA-binding function. All are clearly members of the Hox subgroup (see main text), and the Shx genes are the product of duplication of an ancestral zen/paralogy group 3 Hox gene during the evolution of lepidopterans (see main text). Closely related proteins for which high quality homeodomain tertiary structures have experimentally resolved include two insect Hox proteins (Antp and Ubx), a vertebrate Hox protein (Hoxb1) and a ParaHox protein (Pdx1, not present in insects). The relevant Protein databank codes are: 9ant (crystal structure of *Drosophila* Antp bound to DNA), 1ahd (NMR structure of *Drosophila* Antp bound to DNA), 1b72 (crystal structure of human HOXB1 in ternary complex with PBX1 and DNA), 1b8i (crystal structure of *Drosophila* Ubx in ternary complex with Exd and DNA) and 2h1k (crystal structure of hamster Pdx1 bound to DNA). Preliminary modelling indicated that Antp, Ubx and Pdx1 would be equally good templates for ShxB, ShxC and ShxD, Hoxb1 is a slightly better model for ShxA, and Pdx1 is a slightly better template for zen. For further analysis, structural models of Antp were preferred since this gene is closer evolutionarily than Ubx or Pdx1, and using a single template introduces less variation into the calculations described below. Of the two Antp models, 9ant (chain A) was preferred over the identical sequence of 1ahd (chain A) to remove the contribution of the variable amino terminus (4 residues).

	zen	shxA	shxB	shxC	shxD
9antA	0.39/56 68	0.33/56 57	0.18/56 46	0.30/56 50	0.74/56 37
1ahdA	2.59/60 67	1.56/60 57	3.22/60 47	2.87/60 50	2.63/60 38
1aplA	1.56/55 35	1.56/55 33	1.57/55 33	1.62/55 33	1.20/55 35
1b72A	0.81/57 60	0.89/57 54	0.48/57 49	0.46/57 51	1.22/57 40
1b8iA	0.78/56 64	0.96/56 55	0.85/56 46	0.76/56 50	1.26/56 38
1du0A	0.56/53 53	0.53/53 51	0.47/53 36	0.49/53 40	0.61/53 32
1cqtA	2.11/59 31	2.67/59 24	1.13/59 27	1.73/59 34	1.90/59 32
2gloA	5.19/56 16	5.27/56 14	5.16 57 18	5.26 57 12	5.24/57 19
1au7A	6.26/41 12	4.32/41 12	5.81 41 12	6.13 41 5	5.06/41 15

col.s: RMSD/N %ID = RMSD over N matched residues with % sequence ID

3 Molecular Modelling

Each sequence was modelled on the 9ant chain A structure (without DNA) using the molecular modelling program MODELLER-9v7 [Marti-Renom et al., 2000] which makes the required amino acid substitutions and refines the resulting model. As there were no insertions or deletion of sequence, the modelling exercise was straightforward and the internal energies calculated by MODELLER were taken as a guide to the quality of the model. These included the sum of pairwise atomic interactions (pair), the estimated solvent interaction term (surface) and the overall energy (combined).

The final energies of the models were respectively compared to that of the native sequence of 9ant modelled on itself and to that of 1b72A (human HOXB1) modelled on 9ant. The 1b72A sequence has a similar degree of divergence from the 9ant sequence as the lepidopteran sequences in the current study.

To assess the degree of structural deviation of the models from other homeodomain structures, a root-mean-square deviation (RMSD) was calculated between sets of matched a-carbon atoms. The degree of sequence similarity (percentage amino acid identity, %ID) measured over these superpositions shows that no alternative template is preferable.

```
9antA:   Native energy (pair)     :  -123.376
9antA:   Native energy (surface)  :     0.153
9antA:   Native energy (combined) :   -10.345

shxA:    Native energy (pair)     :  -130.431
shxA:    Native energy (surface)  :    -1.448
shxA:    Native energy (combined) :   -11.731

shxB:    Native energy (pair)     :  -140.319
shxB:    Native energy (surface)  :    -1.592
shxB:    Native energy (combined) :   -11.963

shxC:    Native energy (pair)     :  -131.120
shxC:    Native energy (surface)  :    -4.170
shxC:    Native energy (combined) :   -13.179

shxD:    Native energy (pair)     :  -132.007
shxD:    Native energy (surface)  :    -3.312
shxD:    Native energy (combined) :   -12.741

zen:     Native energy (pair)     :  -134.902
zen:     Native energy (surface)  :    -0.532
zen:     Native energy (combined) :   -10.086

1b72A:   Native energy (pair)     :  -124.436
1b72A:   Native energy (surface)  :    -1.939
1b72A:   Native energy (combined) :   -11.365
```

4 DNA Binding

To assess theoretically whether the lepidopteran Shx homeodomains could bind to DNA, the energy of the protein/DNA complex can be calculated. However as the nature of the DNA sequence to which the proteins bind (if at all) is not known, then a method is required that can test multiple sequences. The Rosetta program [Leaver-Fay et al., 2011] can model protein DNA complexes by applying changes ("mutations") to both the protein and DNA template through a ’resfile’ specification, a typical example of which is shown below.

```
#For all residue positions not specified after ’start’:
NATAA           # allow just native amino acids
EX 1 EX 2 EX 3  # allow extra chi rotameters at chi-id 1, 2 and 3
start
6 A PIKAA T     # change protein position 6 chain A to T (threonine)
7 A PIKAA A
8 A PIKAA F
:
56 A PIKAA E
59 A PIKAA D
60 A PIKAA R
100 B PIKNA T   # change position 100 in DNA chain B to T (thymine)
212 C PIKNA A   # change position 212 in DNA chain C to A (adenine)
101 B PIKNA T
211 C PIKNA A
102 B PIKNA G
:
```

Rosetta can model sequence changes using either a fixed backbone (fast) or a flexible backbone (slow) and models were constructed for each protein sequence initially with the DNA sequence found in the 9ant structure using both methods. To distinguish inter from intra molecular energies, models were constructed with the template protein and DNA in their native bound conformation and with the protein removed to a large distance from the DNA (split). The difference between these configurations should give some indication of the protein/DNA binding energy. Using Rosetta in its relaxed backbone mode the energies of the native (9ant) and all five lepidopteran sequences were of comparable energy and showed a slight trend towards a lower (better) binding energy when the protein/DNA interaction was included. However, an undesirable aspect of the resulting models was a marked distortion in the DNA backbone reaching up to almost 5Å RMSD in some models. To avoid this we turned to a fixed backbone energy calculation.

RELAXED BACKBONE

	9ant	zen	shxA	shxB	shxC	shxD
bound	-128.10	-122.72	-125.43	-120.53	-111.48	-104.38
split	-116.18	-115.44	-120.40	-121.19	-106.12	-103.36
RMSprot(CA)	0.99	0.66	0.51	0.66	0.82	1.12
RMS dna (P)	1.43	3.71	3.40	4.87	3.90	4.93

FIXED BACKBONE (prot and DNA RMS are all 0.0)

	9ant	zen	shxA	shxB	shxC	shxD
bound	-83.48	-73.51	-71.62	7.60	2.21	1397.47
split	-95.55	-85.14	-82.33	-2.51	-9.16	1386.39

PSEUDO-RELAXED PROTEIN BACKBONE

	9ant	zen	shxA	shxB	shxC	shxD
bound	-85.41	-69.74	-67.60	-60.51	-57.23	-51.66
split	-89.23	-83.61	-79.62	-73.10	-74.06	-61.02
RMSprot(CA)	0.07	0.11	0.09	0.12	0.14	0.14

In fixed backbone mode, Rosetta gave reasonable negative energies for the native model and two of the sequences. As would be expected, these were not as low as the energy values with a flexible backbone. The energy of the complex in this mode was now slightly higher, probably as a result of the inability of the DNA to distort and relax poor interactions. However, for three of the interactions, the bound complex had a much higher (positive) energy and for ShxD, this was over 1000. These values remained high in the separated protein/DNA models indicating that they derived from bad intra molecular interactions and not the DNA/protein interface. Detailed examination revealed that they resulted from just a few poor rotamer choices (despite allowing rotamer selection over 3 chi angles) leading to steric clashes within the protein models. Such a large variation in energy would make this mode of operation unsuitable for comparative studies.

While Rosetta allows constraints to be imposed on the degree of flexible backbone refinement, a simpler method was found by introducing a very small amount of noise into the atomic coordinates (in the order of 0.1Å). This biases the choice of rotamer and over multiple runs increasingly lower energy models were found and the best taken as a template. The process is usually converges in 50 iterations but 100 were run for each model. The resulting models show a consistently low energy with minimal RMSD from the starting protein model. (The DNA backbone was kept static).

The energies of the DNA bound and split models now show a consistent level with the bound complex having slightly higher value than the split model which may be a result of the DNA sequence not being optimal for each protein.

5 DNA motif evolution

To find the optimal sequence for each complex, the energies of all possible DNA sequences could be modelled. However, over a 13-mer, this would be a very large number $4^{13}/2$ (not removing palindromes). Alternatively, each of the four bases can be tested individually at each position. This calculation requires only 4´13 models to be evaluated but assumes base-pair independence at each position. Both approaches were used but the full sequence permutation was replaced by random sampling over the 11

core positions with a higher level of sampling in the inner 9 positions. For each DNA sequence considered, the complex was remodelled using Rosetta with the pseudo-relaxed backbone model providing the starting template. This means that all side-chain and base-pair packing was recalculated for each model. To help avoid any anomalous energies like those seen with fixed-backbone repacking, the protein component was given a small rigid-body random displacement (typically 0.1Å) relative to the DNA.

Rosetta employs a generic physico-chemical potential for energy evaluation, of which steric violations contribute the largest component. An alternative approach is to employ an empirical potential that is derived from known examples of a specific type of interaction. One of the more successful empirical potentials for protein/DNA recognition is based on the DFIRE program [Xu et al., 2013]. We used the more recent version specified as DDNA31 [Zhao et al., 2010] (referred to as dFIRE3 below). Both Rosetta and dFIRE3 were used to evaluate alternative sequences by substituting each base at each position. With its greater sensitivity to steric violation, the Rosetta method tends to exclude mutations that result in poor packing whereas dFIRE3 tends to identify preferred interactions. Given the markedly different selection behaviour of the two programs, both were used by taking a combination of their scores as a simple product, with its negative value being minimised.

For each protein, the two programs were used to evaluate 1000 DNA sequences with an average of 4 random substitutions. These results were summarised by ranking the models by score and compiling a frequency motif of the top 50 ranked DNA sequences. For the 9 core positions, there was now clear retention of the core ATTA motif and to a lesser extent the flanking 5' AG and 3' G bases. This analysis provides a measure of the robustness of the starting sequence to mutation but does not give a clear indication of whether each of the proteins has a distinct preferred motif of their own.

To remove the starting bias of the 9ant DNA sequence, the mutation process was started with each of the four homo-polymers (poly-A, -T, -C, -G) and to aid selection of the optimum sequence, the best (lowest scoring) sequence on every cycle was selected for mutation. We applied this protocol to the closely related *P. aegeria* (Speckled Wood) Shx and zen proteins, *P. c-album* (Comma) Shx and zen proteins and to the more distantly related *C. ohridella* (Horse Chestnut Leafminer moth). As a control, we also ran the *Drosophila* Antp homeodomain structure (9ant).

The 4000 sequences generated for each protein were ranked by their score and the bases found at each position were then sorted by type creating a (horizontal) bar-chart of base preference. (These are displayed a sequence-logos in the main text). To the left of each position, the sequence found in 9ant is shown along with a summary of the consensus motif with bases in upper-case if they are present in more than 80% of the sequence and lower-case if more than 40% (taking the most frequent, if more than one base qualifies). To the right of each position, a single character indicates the consensus (majority) base type.

For the *Drosophila* Antp homeodomain 9ant complex, the base frequencies over the 50 best sequences regenerated a reasonable approximation of the known motif. Over the 9 core positions, the ATTA motif is strongly regenerated but with a 20% chance to accept a C in the second T position. In the flanking regions, a G is regenerated in the 5' segment but otherwise the bases in these regions differ from the sequence in 9ant (chain A). To test the robustness of this recapitulation, the same protocol was rerun 12 times and the resulting means and standard deviations of each base frequency are shown to the right of the histogram above. Deviations range from zero up to ten in some of the least constrained positions.

ANTENNAPEDIA

9ant

```
A   AAAACCCCCCCCCCCGGGGTTTTTTTTTTTTTTTTTTTTTTTTTTTTTTT T A = 14.00 6.77  C =  6.17 4.52  G = 10.67  6.39  T = 19.17 7.48
A   AACCCGGGGGGGGGGGGGGGGGGGGGGGGGGGGGGGGGGTTTTTTTTTT G A =  2.33 1.84  C =  1.17 1.95  G = 15.08 10.10  T = 31.42 9.95
A G AAGGGGGGGGGGGGGGGGGGGGGGGGGGGGGGGGGGGGGGGGGGTTTTTT G A =  3.92 4.48  C =  2.83 3.24  G = 33.75  8.95  T =  9.50 8.19
G g ACGGGGGGGGGGGGGGGGGGGGGGGGGGGGGGGGGGGGGGTTTTTTTTT G A =  2.50 1.44  C =  4.25 2.38  G = 27.75  8.27  T = 15.50 7.31
C a AAAAAAAAAAAAAAAAAAAAAAAAAAAAAAAAAAAAAAAACCGGGGGGGG A A = 34.67 4.01  C =  0.17 0.37  G = 14.50  3.93  T =  0.67 1.18
C a AAAAAAAAAAAAAAAAAAAAAAACCCCCCCCCCCCCCCCCCCCGGGGTTT A A = 10.92 6.02  C = 33.58 6.80  G =  3.50  2.18  T =  2.00 1.53
A A AAAAAAAAAAAAAAAAAAAAAAAAAAAAAAAAAAAAAAAAAAAAAAAAAA A A = 48.33 1.37  C =  0.08 0.28  G =  1.58  1.38  T =  0.00 0.00
T T TTTTTTTTTTTTTTTTTTTTTTTTTTTTTTTTTTTTTTTTTTTTTTTTTT T A =  0.00 0.00  C =  0.00 0.00  G =  0.00  0.00  T = 50.00 0.00
T t CCCCCCCCCCGTTTTTTTTTTTTTTTTTTTTTTTTTTTTTTTTTTTTTTT T A =  0.00 0.00  C = 15.58 8.59  G =  0.67  1.18  T = 33.75 9.27
A A AAAAAAAAAAAAAAAAAAAAAAAAAAAAAAAAAAAAAAAAAAAAAAAGTT A A = 48.17 1.82  C =  0.00 0.00  G =  0.42  0.64  T =  1.42 1.85
G t AAAAAAAAAAAAAAAACCCGGGGTTTTTTTTTTTTTTTTTTTTTTTTTTT T A = 21.50 7.57  C =  1.17 0.99  G =  4.08  3.90  T = 23.25 9.43
A   GGGGGGGGGGGTTTTTTTTTTTTTTTTTTTTTTTTTTTTTTTTTTTTTTT T A =  8.08 7.79  C =  0.58 0.76  G =  9.25  7.12  T = 32.08 9.24
G   AAAACCCCCCCCCCCGGGGTTTTTTTTTTTTTTTTTTTTTTTTTTTTTTTT T A = 14.00 6.77  C =  6.17 4.52  G = 10.67  6.39  T = 19.17 7.48
```

shxA

```
A . AAAAAAAAAAAAAAAAAAAAAAAAACCCCCCGGGGGGGTTTTTTTTTTTT A        A . AACCCCCCCCCCCGGTTTTTTTTTTTTTTTTTTTTTTTTTTTTTTTTTTT T
A . CCCCCCCCCCCCCCCCCCCGGGGTTTTTTTTTTTTTTTTTTTTTTTTTTT T        A . AACGGTTTTTTTTTTTTTTTTTTTTTTTTTTTTTTTTTTTTTTTTTTTTT T
A g AAAAAACGGGGGGGGGGGGGGGGGGGGGGGGGGGGGGTTTTTTTTTTTTT G        A G AAACGGGGGGGGGGGGGGGGGGGGGGGGGGGGGGGGGGGGGGGGGGGTTT G
G g AACCCCGGGGGGGGGGGGGGGGGGGGGGGGGGGGGGGGGGGGGGTTTTTT G        G g CCCCCCCCGGGGGGGGGGGGGGGGGGGGGGGGGGGGGGTTTTTTTTTTTT G
C a AAAAAAAAAAAAAAAAAAAAAAAAAAAAAAAAACCGGGGGGGGGGGGGGG A        C g AAAAAAAAAAAAAACCCGGGGGGGGGGGGGGGGGGGGGGGGGGGGGGGGG G
C g AAAAAACCCCCCGGGGGGGGGGGGGGGGGGGGGGGGGGGGGGTTTTTTTT G        C g AAAAAAACCCGGGGGGGGGGGGGGGGGGGGGGGGGGGGGGGGTTTTTTTT G
A A AAAAAAAAAAAAAAAAAAAAAAAAAAAAAAAAAAAAAAAAAAAAAAAAAA A        A A AAAAAAAAAAAAAAAAAAAAAAAAAAAAAAAAAAAAAAAAAAAAAAAAAA A
T T TTTTTTTTTTTTTTTTTTTTTTTTTTTTTTTTTTTTTTTTTTTTTTTTTT T        T T TTTTTTTTTTTTTTTTTTTTTTTTTTTTTTTTTTTTTTTTTTTTTTTTTT T
T t CCCCCCCCCCCCCCCCTTTTTTTTTTTTTTTTTTTTTTTTTTTTTTTTTT T        T c CCCCCCCCCCCCCCCCCCCCCCCCCCCCCCCCCCCCCTTTTTTTTTTTTT C
A A AAAAAAAAAAAAAAAAAAAAAAAAAAAAAAAAAAAAAAAAAAAAAAAAAA A        A A AAAAAAAAAAAAAAAAAAAAAAAAAAAAAAAAAAAAAAAAAAAAAAAAAA A
G g AAAAAAAAAAAAAAAGGGGGGGGGGGGGGGGGGGGGGGGGGGGGGGGGGG G        G A AAAAAAAAAAAAAAAAAAAAAAAAAAAAAAAAAAAAAAAAAAAGGGGTTT A
A . ACCCCCCCCCCCGGGGGGGGGGGGGGGGGGGGGGGGGGGTTTTTTTTTTT G        A . AAAGTTTTTTTTTTTTTTTTTTTTTTTTTTTTTTTTTTTTTTTTTTTTTT T
G . AAAAAAAAAAAAAAAAAAAAAAAAACCCCCCGGGGGGGTTTTTTTTTTTT A        G . AACCCCCCCCCCCGGTTTTTTTTTTTTTTTTTTTTTTTTTTTTTTTTTTT T
```

shxB

```
A . AAAAAAAAAAAACCCCCCCGGGGGGGGGGGGGGGGGGGTTTTTTTTTTTT G        A . AAAAAAAAAAAAAACCCCCGGGGGGGGGGGGGGGGGGGGTTTTTTTTTTT G
A . AAAAAAAAAAACGGGGGGGGGGGGGGGGGGGGGTTTTTTTTTTTTTTTTT T        A . AACCCCCCCCCCCCCCCCCCCCCCCCCCCCCCCCGGGGGGGGGGGGGGGG C
A g AAAAAAAAAAAAAAAAACCCCCCCCCGGGGGGGGGGGGGGGGGGGGTTTT G        A c ACCCCCCCCCCCCCCCCCCCCGGGGGGGGGGGGGGGGGGGGTTTTTTTTT C
G g AACCCCCCCCCCCCGGGGGGGGGGGGGGGGGGGGGGTTTTTTTTTTTTTT G        G c CCCCCCCCCCCCCCCCCCCCCCCGGGGGGGGGGTTTTTTTTTTTTTTTTT C
C a AAAAAAAAAAAAAAAAAAAAAAAAAAAACCCCCCGGGGGGGGGGGGGGGG A        C a AAAAAAAAAAAAAAAAAAAAAAAAAAAAAAAACGGGGGGGGGGGGGGGGG A
C G AAAAACCGGGGGGGGGGGGGGGGGGGGGGGGGGGGGGGGGGGGGGGGGGG G        C G AAAGGGGGGGGGGGGGGGGGGGGGGGGGGGGGGGGGGGGGGGGGGGGGGG G
A A AAAAAAAAAAAAAAAAAAAAAAAAAAAAAAAAAAAAAAAAAAAAAAAAAA A        A A AAAAAAAAAAAAAAAAAAAAAAAAAAAAAAAAAAAAAAAAAAAAAAAAAG A
T T TTTTTTTTTTTTTTTTTTTTTTTTTTTTTTTTTTTTTTTTTTTTTTTTTT T        T T TTTTTTTTTTTTTTTTTTTTTTTTTTTTTTTTTTTTTTTTTTTTTTTTTT T
T t CCCCCCCCCCCCCCCCGTTTTTTTTTTTTTTTTTTTTTTTTTTTTTTTTT T        T c CCCCCCCCCCCCCCCCCCCCCCCCCCCCTTTTTTTTTTTTTTTTTTTTTT C
A A AAAAAAAAAAAAAAAAAAAAAAAAAAAAAAAAAAAAAAAAAAGGGGGTTT A        A A AAAAAAAAAAAAAAAAAAAAAAAAAAAAAAAAAAAAAAAAAAAAAAAAAG A
G t AAAAAAAAAAGGGGGGGGGGGGGGTTTTTTTTTTTTTTTTTTTTTTTTTT T        G a AAAAAAAAAAAAAAAAAAAAAAAAAAAAAAAACCCCCGGGTTTTTTTTTT A
A . AACCCCCCCCCCCGGGGTTTTTTTTTTTTTTTTTTTTTTTTTTTTTTTTT T        A . AAAAAAAAAACGGGGGGGGGGGGGGGGGGGGGGGGGGGGGTTTTTTTTTT G
G . AAAAAAAAAAAACCCCCCCGGGGGGGGGGGGGGGGGGGTTTTTTTTTTTT G        G . AAAAAAAAAAAAAACCCCCGGGGGGGGGGGGGGGGGGGGTTTTTTTTTTT G
```

shxC

```
A . AAAAAAAAAAAAAAAACCCGGGGGGGGGGGGGGGGGGGGTTTTTTTTTTT G        A . AAAAAAAAAAACCCCCCCCCCCCCGGGGGTTTTTTTTTTTTTTTTTTTTT T
A . AAAAAAAAAAAAAACCCCCCCCCCCCCGGGGGGGGGGGGGGGGGGGGTTT G        A . ACCCCCCCCCCCCCCCCCCCCCCCCCGGGGGGGTTTTTTTTTTTTTTTTT C
A c AAAAAAAAACCCCCCCCCCCCCCCCCCCCCCCCCCCCCCCCCCCGGGGGGT C        A a AAAAAAAAAAAAAAAAAAAAAAAAAACCCCCCCCCCCCCCGGGTTTTTTT A
G t ACCCCCCCGGGGGGGGGGGGGGGTTTTTTTTTTTTTTTTTTTTTTTTTTT T        G T ACCGGGGGTTTTTTTTTTTTTTTTTTTTTTTTTTTTTTTTTTTTTTTTTT T
C g AAAAAAAAAAAAAAAAACCGGGGGGGGGGGGGGGGGGGGGGGGGGGGGGG G        C g AAAAAAAAAAAAAACGGGGGGGGGGGGGGGGGGGGGGGGGGGGGGGGGGG G
C G CCCCCGGGGGGGGGGGGGGGGGGGGGGGGGGGGGGGGGGGGGGGGGGGGG G        C G CCCGGGGGGGGGGGGGGGGGGGGGGGGGGGGGGGGGGGGGGGGGGGGGGG G
A A AAAAAAAAAAAAAAAAAAAAAAAAAAAAAAAAAAAAAAAAAAAAAAAAAA A        A A AAAAAAAAAAAAAAAAAAAAAAAAAAAAAAAAAAAAAAAAAAAAAAAAAA A
T T TTTTTTTTTTTTTTTTTTTTTTTTTTTTTTTTTTTTTTTTTTTTTTTTTT T        T T TTTTTTTTTTTTTTTTTTTTTTTTTTTTTTTTTTTTTTTTTTTTTTTTTT T
T c CCCCCCCCCCCCCCCCCCCCCCCCCCCCCCCCCCCCCCTTTTTTTTTTTT C        T t CCCCCCCCCCCCCCCCCCCTTTTTTTTTTTTTTTTTTTTTTTTTTTTTTT T
A A AAAAAAAAAAAAAAAAAAAAAAAAAAAAAAAAAAAAAAAAAAAAAAAAAA A        A A AAAAAAAAAAAAAAAAAAAAAAAAAAAAAAAAAAAAAAAAAAATTTTTTT A
G a AAAAAAAAAAAAAAAAAAAAAAAAAAACCGGGTTTTTTTTTTTTTTTTTT A        G a AAAAAAAAAAAAAAAAAAAAAAAGGGGGGGGGGGGGGGGGGTTTTTTTTT A
A . AAGGGGGGGGGGGGGGGGGGGGGGGGGGGGGGGGGGGGGGGGGGGGGTTT G        A . AGGGGGGGGGGGGGGGGGGTTTTTTTTTTTTTTTTTTTTTTTTTTTTTTT T
G . AAAAAAAAAAAAAAAACCCGGGGGGGGGGGGGGGGGGGGTTTTTTTTTTT G        G . AAAAAAAAAAACCCCCCCCCCCCCGGGGGTTTTTTTTTTTTTTTTTTTTT T
```

shxD

```
A . ACCCCCGGGGGGGGGGGGGGTTTTTTTTTTTTTTTTTTTTTTTTTTTTTT T        A . AAAAAAACCCCCGGGGGGGGGGGGGGGGGGGGTTTTTTTTTTTTTTTTTT G
A . AAAAAACCCGGGGGGGGGGGGGGTTTTTTTTTTTTTTTTTTTTTTTTTTT T        A . CCCCCCCCCCCCCCCCCCCCCCCCCCCCCCCCCCCCCCCCCGTTTTTTTT C
A C CCCCCCCCCCCCCCCCCCCCCCCCCCCCCCCCCCCCCCCCCCCGGGGGGG C        A c AAAAAAAAAAAAAAAACCCCCCCCCCCCCCCCCCCCCCCCCCGGGGGGGT C
G g GGGGGGGGGGGGGGGGGGGGGGGGGGGGGGGGTTTTTTTTTTTTTTTTTT G        G t CCCCCCGGGGGGGGGGGTTTTTTTTTTTTTTTTTTTTTTTTTTTTTTTTT T
C G AAAAAACGGGGGGGGGGGGGGGGGGGGGGGGGGGGGGGGGGGGGGGGGGG G        C g AAAAAAAAAAAAAACCCCCCCCCCGGGGGGGGGGGGGGGGGGGGGGGGGG G
C T AGTTTTTTTTTTTTTTTTTTTTTTTTTTTTTTTTTTTTTTTTTTTTTTTT T        C T CCCTTTTTTTTTTTTTTTTTTTTTTTTTTTTTTTTTTTTTTTTTTTTTTT T
A A AAAAAAAAAAAAAAAAAAAAAAAAAAAAAAAAAAAAAAAAAAAAAAAAAC A        A A AAAAAAAAAAAAAAAAAAAAAAAAAAAAAAAAAAAAAAAAAAAAAAAAAA A
T T TTTTTTTTTTTTTTTTTTTTTTTTTTTTTTTTTTTTTTTTTTTTTTTTTT T        T T TTTTTTTTTTTTTTTTTTTTTTTTTTTTTTTTTTTTTTTTTTTTTTTTTT T
T T CCCCCCCCCTTTTTTTTTTTTTTTTTTTTTTTTTTTTTTTTTTTTTTTTT T        T T CCCCCCCCTTTTTTTTTTTTTTTTTTTTTTTTTTTTTTTTTTTTTTTTTT T
A A AAAAAAAAAAAAAAAAAAAAAAAAAAAAAAAAAAAAAAAAAAAAAAAAAA A        A t AAAAAAAAAAAACCCCCGGTTTTTTTTTTTTTTTTTTTTTTTTTTTTTTT T
G A AAAAAAAAAAAAAAAAAAAAAAAAAAAAAAAAAAAAAAAAAAAACGGGTT A        G a AAAAAAAAAAAAAAAAAAAAAAAAAAAAAAAAGGGGGGGGGGTTTTTTTT A
A . ACCGGGGGGGGGGGGGGGGGGTTTTTTTTTTTTTTTTTTTTTTTTTTTTT T        A . AAGGGGGGGGGGGGGGGGGGGGGGGGGGGGTTTTTTTTTTTTTTTTTTTT G
G . ACCCCCGGGGGGGGGGGGGGTTTTTTTTTTTTTTTTTTTTTTTTTTTTTT T        G . AAAAAAACCCCCGGGGGGGGGGGGGGGGGGGGTTTTTTTTTTTTTTTTTT G
```

Zen

```
  . AAAAAAAACCCCCCCGGGGGGGGGGGGGGTTTTTTTTTTTTTTTTTTTTT T        A . AAAAAAAACCCCCCCCCCCCCCGGGGGGGGGGGGGTTTTTTTTTTTTTTT G
A . AAAAAAAAGGGGGGGGGGGGGGGGGGGGGGGGTTTTTTTTTTTTTTTTTT G        A . AAAAAAAAAAAAAAAAAAAAAAAAGGGGGGGGGGGGGGGGGGGGGGGGTT G
A g AACGGGGGGGGGGGGGGGGGGGGGGGGGGGGGGGGGGGGGGGGTTTTTTT G        A g AAAACCCCCCGGGGGGGGGGGGGGGGGGGGGGGGGGGTTTTTTTTTTTTT G
G t AAACGGGGGGGGGGGGGGGGGGGGGTTTTTTTTTTTTTTTTTTTTTTTTT T        G g AAACCGGGGGGGGGGGGGGGGGGGGGGGGGGGGGGGGGGGGGTTTTTTTT G
C g AAAAAAAAAAAAAAAAAAAGGGGGGGGGGGGGGGGGGGGGGGGGGGGGGG G        C g AAAAAAAAAAAAAAAAAGGGGGGGGGGGGGGGGGGGGGGGGGGGGGGGGG G
C A AAAAAAAAAAAAAAAAAAAAAAAAAAAAAAAAAAAAAAAAAAGGGGGGGG A        C A AAAAAAAAAAAAAAAAAAAAAAAAAAAAAAAAAAAAAAAAAAAAGGGGGG A
A A AAAAAAAAAAAAAAAAAAAAAAAAAAAAAAAAAAAAAAAAAAAAAAAAAG A        A A AAAAAAAAAAAAAAAAAAAAAAAAAAAAAAAAAAAAAAAAAAAAAAAAAA A
T T TTTTTTTTTTTTTTTTTTTTTTTTTTTTTTTTTTTTTTTTTTTTTTTTTT T        T T TTTTTTTTTTTTTTTTTTTTTTTTTTTTTTTTTTTTTTTTTTTTTTTTTT T
T c CCCCCCCCCCCCCCCCCCCCCCCCCGTTTTTTTTTTTTTTTTTTTTTTTT C        T t CCCCCCCCCCCCCCCCCCCTTTTTTTTTTTTTTTTTTTTTTTTTTTTTTT T
A a AAAAAAAAAAAAAAAAAAAAAACCGTTTTTTTTTTTTTTTTTTTTTTTTT A        A t AAAAAAAAAACCGGGGGGGTTTTTTTTTTTTTTTTTTTTTTTTTTTTTTT T
G G AAAAAGGGGGGGGGGGGGGGGGGGGGGGGGGGGGGGGGGGGGGGGGGGTT G        G a AAAAAAAAAAAAAAAAAAAAAAAAAAAAAAAAAGGGGGGGGTTTTTTTTT A
A . GGGGGGGGGGGGGGGGGGGGGGGGGTTTTTTTTTTTTTTTTTTTTTTTTT G        A . AAAGGGGGGGGGGGGGGGGGGTTTTTTTTTTTTTTTTTTTTTTTTTTTTT T
G . AAAAAAAACCCCCCCGGGGGGGGGGGGGGTTTTTTTTTTTTTTTTTTTTT T        G . AAAAAAAACCCCCCCCCCCCCCGGGGGGGGGGGGGTTTTTTTTTTTTTTT G
```

The consensus motifs for Comma were similar to the *Drosophila* results but with an increased trend to accept a C in the second T position in the core ATTA motif, to the extent that in two sequences, ShxC and zen, C was in the majority. In the zen sequence, a propensity to accept a T in the second A position in the motif also emerged. In the regions flanking the core, some strong selections were observed but these tended to differ from the sequence in the Antp homeodomain complex. Compared to the similar Speckled Wood sequences, the only marked difference was the strong preference for a G in the first position of the core motif in the Speckled Wood ShxD sequence (which was retained over repeated trials). The Speckled Wood ShxD sequence differs from Comma at a core position (47) that packs against the complement to this base (C). From an examination of the structures, a possible explanation why the VAL to ILE change should favour a G over A is that the T complement of the A has its methyl group pointing towards the ILE which may result in unfavourable steric hindrance. Some test mutations partly confirmed this conclusion but also indicated that the situation was more complex than a single residue/base interaction.

The sequences of the Leafminer moth exhibited the same trends seen in Comma with the core motif AT[TC]A being a better summary. The possibility of a T in the second A position was now seen to be the prefered base not only in zen but also in ShxD.

As the terminal positions of the 13-mer are not mutated, it can be seen from their base frequency that the majority of the good solutions derive from the poly-T starting sequence. (Most probably because of a selective advantage of having the core TT motif sequence from the beginning). This may introduce a T bias into other positions and to allow for this, each sequence was mutated (without evolution) starting from their consensus sequence with mutations extended to cover the full 13-mer equally.

regenerated consensus motifs

	EVOLVED	RESTART	REFINED (top 50 consensus)	
			AA-AGCCATTAG-AG	9antA (known)
9ant	GgaaATtAt	TGGGACATTATTT	tg-GGACATTAt-Tt	
ShxA	ggagATtAg	ATGGAGATTAGGA	aT-gGAGATTAG-ga	
ShxB	ggaGATtAt	GTGGAGATTATTG	gt-GgAGATTAt-Tg	
ShxC	ctgGATcAa	GGCTGGATCAAGG	gg-CtGGATCAA-Gg	
ShxD	CgGTATTAA	TTCGGTATTAATT	tT-CGGTATTAA-TT	
Zen	gtgAATcaG	TGGTGAATCAGGT	.g-gTGAATcag-gT	

The consensus over the top 50 scoring sequences after refinement generally reaffirms the original evolved consensus sequence taken as a starting position with no changes (except in emphasis) being seen over the core 9 positions. In the flanking termini, a few position shift their base preference but as there is little selection pressure at the termini, the identity of the starting base is generally retained.

6 Conclusions

Under all the tests, the five proteins in both species behave as if they are competent and functional homeodomains and, without any starting bias, are able to recapitulate the core ATTA motif in their bound DNA, with a few exceptions where an ATCA motif is preferred. There is more extensive variation in the positions flanking this core and whether these are significant is not easy to assess and will require more extensive analysis.

References

[Leaver-Fay et al., 2011] Leaver-Fay, A., Tyka, M., Lewis, S., Lange, O., Thompson, J., Jacak, R., Kaufman, K., Renfrew, P., Smith, C., Sheffler, W., Davis, I., Cooper, S., Treuille, A., Mandell, D., Richter, F., Ban, Y., Fleishman, S., Corn, J., Kim, D., Lyskov, S., Berrondo, M., Mentzer, S., Popovi, Z., Havranek, J., Karanicolas, J., Das, R., Meiler, J., Kortemme, T., Gray, J., Kuhlman, B., Baker, D. & Bradley, P. (2011). ROSETTA3: an object-oriented software suite for the simulation and design of macromolecules.
Methods Enzymol. 487, 545–574.
http://www.rosettacommons.org/manuals/archive/rosetta3.4 user guide.

[Marti-Renom et al., 2000] Marti-Renom, M. A., Stuart, A., Fiser, A., Sanchez, R., Melo, F. & Sali, A. (2000). Comparative protein structure modeling of genes and genomes. Annu. Rev. Biophys. Biomol. Struct. 29, 291–325.
http://salilab.org/modeller/.

[Xu et al., 2013] Xu, B., Schones, D. E., Wang, Y., Liang, H. & Li, G. (2013). A structural-based strategy for recognition of transcription factor binding sites. PLoS1, 8, e52460.

[Zhao et al., 2010] Zhao, H., Yang, Y. & Zhou, Y. (2010). Structure-based prediction of DNA-binding proteins by structural alignment and a volume-fraction corrected DFIRE-based energy function. Bioinformatics, 26, 1857–1863.
http://sparks.informatics.iupui.edu/yueyang/DFIRE/ddna3-DB-service.
