# Supplementary material for: Ancient Expansion of the Hox Cluster in Lepidoptera Generated Four Homeobox Genes Implicated in Extra-Embryonic Tissue Formation
Source: PLoS Genet. 2014 Oct 23;10(10):e1004698. doi: 10.1371/journal.pgen.1004698 (PMC4207634; doi:10.1371/journal.pgen.1004698)
Supplement: Note S2 — Whole Mount in situ Hybridisation on Pararge aegeria ovarioles and embryos. (DOCX) [file pgen.1004698.s016.docx]

# Formaldehyde Fixation of *Pararge aegeria* ovarioles and embryos

# Ovary Fixation

### Precool PBS on ice.

## Dissect Ovaries out in ice cold **PBS**.

## Rinse to remove debris in ice cold **PBS**.

## Transfer to a 1:1 mixture of **5%FA** + Heptane.

## Fix overnight with gentle rotation.

## Wash three times, 5 minutes each in **PTW** with gentle rotation.

## Dehydrate the tissue:

### 5 minutes in PBS

### 5 minutes in 10% methanol/PBS

### 5 minutes in 25% methanol/PBS

### 5 minutes in 50% methanol/PBS

### 5 minutes in 75% methanol/PBS

### 5 minutes in 90% methanol/PBS

## Fix in 100% methanol for a few days at –20°C.

# Embryo Fixation

## Collect eggs of desired age.

## In a small glass pot, dechorionate the embryos with 50% bleach (4% NaClO) for 1–2 minutes.

## Dilute with water if the reaction is getting too aggressive.

## Rinse well with water to remove any bleach.

## Transfer to a 1:1 mixture of **5%FA** + Heptane.

## Fix overnight with gentle rotation.

## Wash three times, 5 minutes each in **PTW** with gentle rotation.

## Dehydrate the tissue:

### 5 minutes in PBS

### 5 minutes in 10% methanol/PBS

### 5 minutes in 25% methanol/PBS

### 5 minutes in 50% methanol/PBS

### 5 minutes in 75% methanol/PBS

### 5 minutes in 90% methanol/PBS

## Fix in 100% methanol for a few days at –20°C.

# Whole Mount *in situ* hybridisation on *Pararge aegeria* ovarioles and embryos

# Rehydration:

### 5 min per wash in: 90%; 75%; 50%; 25%; 10% methanol:PBS.

### 5 minutes in **PTW**

## Optional: Dissect in **PTW** if necessary.

## Wash 3x 5min in **PTW**.

# Pre-Hybridization

## Digest for 12 min in ice cold **PTW**-**K**.

## Rinse twice in **PTW-G** to stop digest.

## Wash 2x 5min in **PTW**.

## PostFix for 15min in fixative **5%FA**.

## Wash 3x 5min in **PTW**.

## Wash 2x 10min in **PTW**.

## Wash 1x 5min in 1:1 mixture of **PTW**:**preHB**.

## Incubate in **preHB** for 1-2H at [Tm-5]ºC with rocking.

# HB+denatured Probe solution (**HBdP**) preparation (during incubation)

## Denature Yeast tRNA 5min at 80ºC and make **HB** solution.

## Add 1μl of 50–100 ng/μl RNA probe to 100μl of **HB** to make **HBP**.

## Heat-denature **HBP** 5min at 80°C then hold at 70°C.

## Quickly add 900μl warm **HB** to make **HBdP**.

### Prepare both a sense (control) probe and an antisense probe for each target transcript.

# Hybridization

## Incubate sample immediately in **HBdP** overnight at [Tm-5]°C with rocking.

# Post-Hybridization

## Wash 3x 5min in warmed **preHB** at [Tm-5]°C.

## Wash 2x 20min in warmed **preHB** at [Tm-5]°C.

## Allow to settle at room temperature.

## Wash 1x 5min in 1:1 mixture of **PTW:preHB**.

## Wash 2x 5min in **PTW**.

## Incubate in 1x **BLR** (Roche) for 30min with rocking.

## Incubate in **PTW-AB** with rocking for 3-4H at 25°C.

# Pre-Staining

## Wash 3x 5min in **PTW**

## Wash 2x 10min in **PTW**

## Incubate overnight in **PTW**

# Staining

## Wash 2x 5min in **APB** (fresh)

## Stain with **APB+S**.

### Protect from light.

- Optional: Add 1μl/ml **LSS** to **APB+S** to counter endogenous phosphatases.

## When colored, remove the **APB+S**.

## Optional: Add drop of formaldehyde to fix stain.

## Wash 2x 5min in **PTW**.

## Place samples in **PTW** on a glass chamber slide.

## Observe under microscope.

### Jean-Michel Carter, July 2013.

# Abbreviations & Contents

**5%FA 5.5% Formaldehyde**

37% Formaldehyde diluted in 1x PBS.

**APB Alkaline Phosphatase Buffer**

100 mM Tris pH 9.5, 100 mM NaCl, 50 mM MgCl2, 0.1% Tween 20.

**APB+S Alkaline Phosphatase Buffer + Substrate**

100 mM Tris pH 9.5, 100 mM NaCl, 50 mM MgCl2, 0.1% Tween 20 with 1/200 NBT/BCIP.

**BLR Blocking Reagent**

Diluted from 10x solution supplied by Roche Applied Science.

**HB Hybridisation Buffer**

50% Deionized formamide, 5x SSC, 0.02% Tween 20, 100 μg/ml Yeast tRNA, 2 mg/ml Glycine.

**HBdP Hybridisation Buffer with denatured RNA Probe**

50% Deionized formamide, 5x SSC, 0.02% Tween 20, 100 μg/ml Yeast tRNA, 2 mg/ml Glycine with 50–100 ng/μl denatured RNA probe.

**HBP Hybridisation Buffer with RNA Probe**

50% Deionized formamide, 5x SSC, 0.02% Tween 20, 100 μg/ml Yeast tRNA, 2 mg/ml Glycine with 50–100 ng/μl RNA probe.

**LSS Levamisole Stock Solution**

1M in DEPC treated H2O.

**PBS Phosphate Buffered Saline**

Diluted from 10x solution (ThermoFisher Scientific, Waltham, Massachusetts, USA).

**preHB Pre-Hybridisation Buffer**

50% Deionized formamide, 5x SSC, 0.02% Tween 20.

**PTW Phosphate Buffered Saline with Tween 20**

1x PBS, 0.1% Tween 20.

**PTW-G Phosphate Buffered Saline with Tween 20 and Glycine**

1x PBS, 0.1% Tween 20, 2 mg/ml Glycine.

**PTW-K Phosphate Buffered Saline with Tween 20 and Proteinase K**

1x PBS, 0.1% Tween 20, 12.5 μg/ml Proteinase K.

**PTW-AB Phosphate Buffered Saline with Tween 20 and Anti-Digoxigenin-AP**

1x PBS, 0.1% Tween 20 with 1/2000 Anti-Digoxigenin-AP (Roche Applied Science, Penzberg, Germany).
